# Visible‐Light Driven Acetylene Semi‐Hydrogenation to Ethylene With a Copper Photosensitizer and a Cobaloxime Catalyst

**DOI:** 10.1002/chem.71119

**Published:** 2026-05-16

**Authors:** Anna Fortunato, Sharon Silloni, Brando Adranno, Marianna Barbieri, Luka Đorđević, Francesca Arcudi

**Affiliations:** ^1^ Department of Chemical Sciences Università di Padova Padova Italy

## Abstract

The purification of ethylene streams from acetylene impurities remains a key industrial challenge, for which light‐powered strategies are emerging as sustainable alternatives to conventional approaches. Recent studies have shown that hydrogen atom transfer (HAT) pathways enable highly selective acetylene semi‐hydrogenation by ensuring rapid and controlled hydrogen delivery while suppressing over‐hydrogenation or H_2_ evolution. Here, we report a fully noble‐metal‐free homogenous photocatalytic system in which a molecular cobaloxime catalyst [Co(dmgH)_2_(Im)Cl] (dmg = dimethylglyoxime, Im = imidazole) operates in tandem with a Cu‐based photosensitizer [Cu(N^N)(P^P)]^+^ to drive the selective transfer hydrogenation from acetylene to ethylene under visible light irradiation. Under a pure C_2_H_2_ atmosphere, our system converts acetylene to ethylene with a turnover number (TON) of 670 after 48 h of visible‐light irradiation with 99.6% selectivity, while under ethylene‐rich conditions, acetylene impurities are completely removed with 99.3% selectivity. Mechanistic studies reveal that catalysis proceeds via a Co^III^─H intermediate. Overall, this work demonstrates that rational pairing of cobaloxime catalysts with earth‐abundant Cu‐based photosensitizers provides an efficient and sustainable strategy for photocatalytic acetylene semi‐hydrogenation and ethylene purification.

## Graphical abstract

A fully noble‐metal‐free photocatalytic system that couples cobaloxime catalysis with Cu photosensitizer for HAT‐driven acetylene‐to‐ethylene conversion is reported.

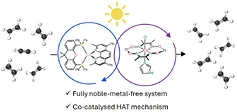

## 1 Introduction

Ethylene is one of the most important commodity chemicals, serving as a key feedstock for the production of ∼50%–60% of all plastics [1]. Industrially, C_2_H_4_ is mainly obtained via steam cracking of petroleum, a process that inevitably generates acetylene impurities. Even trace amounts of acetylene act as poison for Ziegler–Natta polymerization catalysts, making its selective removal or conversion to ethylene crucial for producing polymer‐grade ethylene streams [2, 3, 4, 5]. At present, this purification step relies on a thermocatalytic process using Pd‐based catalysts under high temperatures (100°C–300°C), and an external H_2_ co‐feed, resulting in high energy consumption, elevated costs, and limited selectivity due to over‐hydrogenation [6]. These drawbacks have motivated the search for alternative, more sustainable, and selective catalytic routes, among which photocatalytic acetylene semi‐hydrogenation has recently emerged as a promising strategy [7, 8, 9, 10, 11, 12, 13, 14, 15, 16, 17, 18].

Recent studies by our group [8, 9, 11, 13, 18] and others [7, 10, 12, 14, 15, 16, 17] have demonstrated homogeneous and heterogeneous photocatalytic systems for the selective conversion of acetylene to ethylene under ambient conditions, harnessing solar energy while avoiding the use of molecular H_2_. These systems predominantly rely on Co‐based catalysts, which are particularly well‐suited for this transformation. Specifically, rational catalyst design enables selective ethylene formation via a transition‐metal‐mediated hydrogen atom transfer (HAT) pathway initiated by reactive Co^III^─hydride intermediates, as demonstrated by our groups using cobalt porphyrins [9] and cobaloximes [11], as well as by Dai and coworkers employing Cosalen catalysts [7].

While catalyst structure remains central to achieving high activity and selectivity, the overall photocatalytic performance also critically depends on the nature of the photosensitizer. To date, the majority of reported systems rely on Ru‐ or Ir‐based complexes as benchmark photosensitizers owing to their favorable photophysical properties and redox potentials [19]. However, the scarcity, cost, and sustainability concerns associated with noble‐metal photosensitizers pose significant barriers to large‐scale implementation. Although isolated examples of noble‐metal‐free photosensitizers have begun to appear, their integration with efficient cobalt‐mediated acetylene transfer hydrogenation remains limited and insufficiently explored [12, 13, 16].

Here, we address this gap by expanding the catalyst‐photosensitizer palette for Co‐mediated photocatalytic acetylene transfer semi‐hydrogenation. Cobaloximes represent an attractive catalyst class due to their synthetic accessibility, ligand tunability, and well‐established ability to form catalytically competent Co^III^─H species [20, 21, 22]. In parallel, copper‐based photosensitizers have recently emerged as promising earth‐abundant alternatives to Ru and Ir complexes, offering tunable excited‐state redox properties and visible‐light absorption [23, 24].

**FIGURE 1:**
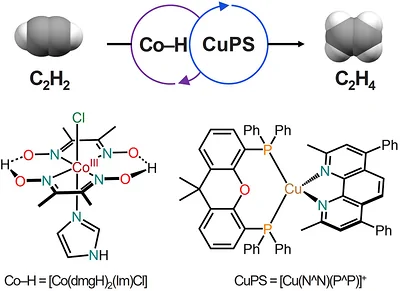
Photocatalytic semi‐hydrogenation of acetylene to ethylene by a cobaloxime‐based catalyst ([Co(dmgH)_2_(Im)Cl]) and a noble‐metal‐free photosensitizer (CuPS).

In this work, we demonstrate that coupling a molecular cobaloxime catalyst [Co(dmgH)_2_(Im)Cl] (dmg = dimethylglyoxime, Im = imidazole) with a Cu‐based photosensitizer [Cu(N^N)(P^P)]^+^ (CuPS) affords a fully noble‐metal‐free photocatalytic system for the semi‐hydrogenation of acetylene to ethylene under visible light irradiation (Figure 1). Operating in an water:acetonitrile (ACN) mixture, the system achieves high activity and selectivity, reaching nearly quantitative acetylene conversion within 105 min of irradiation under ethylene‐rich, industrially relevant conditions. Mechanistic investigations reveal that catalysis proceeds via an efficient Co^III^─H‐mediated HAT pathway, expanding the design landscape of photosensitizers and catalysts and the space for sustainable ethylene purification technologies.

## 2 Results and Discussion

The Cu‐based photosensitizer (CuPS) and the cobaloxime catalyst ([Co(dmgH)_2_(Im)Cl]) were synthesized according to reported procedures (Figures S1–S3) [25, 26, 27, 28, 29]. The photophysical properties of CuPS are consistent with those of related heteroleptic Cu(I) complexes [30, 31]. Its absorption spectrum features a broad band centered at 389 nm (*ɛ* = 5.09 × 10^3^ M^−1^ cm^−1^) attributed to a metal‐to‐ligand charge transfer (MLCT) transition with contributions from interligand charge transfer (ILCT) character (Figure S4) [28, 32, 33].

**FIGURE 2:**
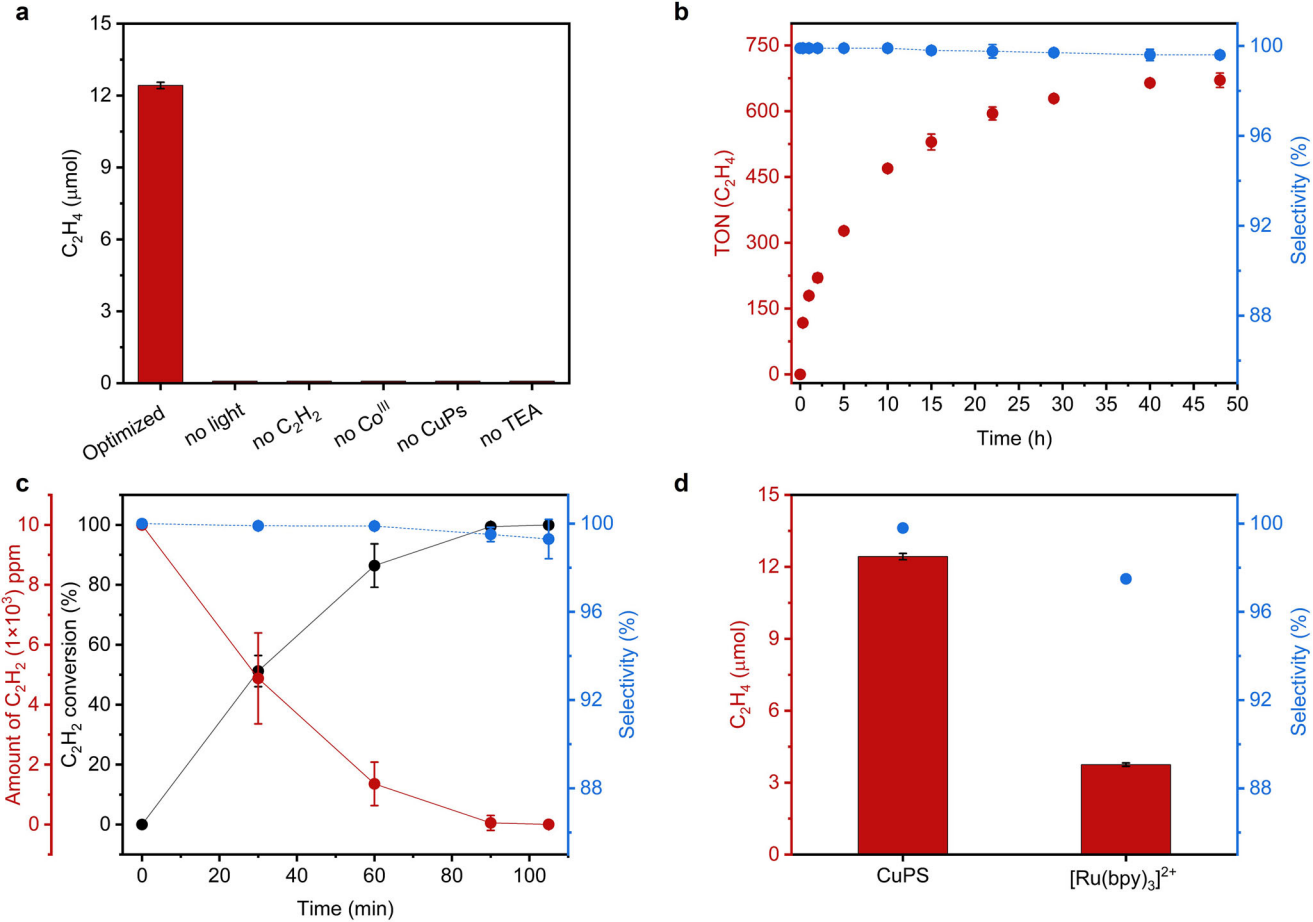
(a) Total C_2_H_4_ production for the optimized reaction mixture containing 100 µM [Co(dmgH)_2_(Im)Cl] and 250 µM CuPS in H_2_O:ACN mixture (1:1 *v/v*) with 10% *v/v* TEA (pH = 11.0 ± 0.3) irradiated (405 nm, 140 mW×cm^−2^) for 4 h under C_2_H_2_ (> 99.5 vol.%), and for reaction mixtures that differ from the optimized conditions as indicated by the axis label. (b) TON (C_2_H_4_) and C_2_H_4_ selectivity (%) over C_2_H_6_ and H_2_ as a function of the irradiation time (405 nm, 140 mW×cm^−2^) for the CuPS/[Co(dmgH)_2_(Im)Cl] system containing 1 µM [Co(dmgH)_2_(Im)Cl] and 250 µM CuPS in H_2_O:ACN mixture (1:1 *v/v*) with 10% *v/v* TEA (pH = 11.0 ± 0.3) under C_2_H_2_ (> 99.5 vol.%). (c) Plot of C_2_H_2_ conversion (%), residual C_2_H_2_ (ppm), and C_2_H_4_ selectivity over C_2_H_6_ (%) as a function of irradiated time under industrially relevant conditions (1 vol.% C_2_H_2_, 30 vol.% C_2_H_4_, N_2_ balance). (d) Total C_2_H_4_ production for reaction mixtures containing 100 µM of catalyst and 250 µM of photosensitizer in a H_2_O:ACN mixture (1:1 *v/v*) with 10% *v/v* of TEA (pH = 11.0 ± 0.3) irradiated for 4 h under C_2_H_2_ (> 99.5 vol.%). Catalytic mixtures containing [Ru(bpy)_3_]^2+^ were irradiated with 450 nm LED (140 mW×cm^−2^), while CuPS‐containing mixtures were irradiated with 405 nm LED (140 mW×cm^−2^).

Photocatalytic C_2_H_2_ transfer hydrogenation was first investigated under non‐competitive conditions by irradiating the catalytic mixture under 1 atm of C_2_H_2_ (> 99.5 vol.%). Experimental details of gas purging and photocatalytic setups (Figure S5) are published elsewhere [8]. The photocatalytic system comprises [Co(dmgH)_2_(Im)Cl] as the catalyst, CuPS as the photosensitizer, and triethylamine (TEA) as the sacrificial donor in a H_2_O:ACN solvent mixture. Product analysis was performed by gas chromatography using both a thermal conductivity detector (TCD) and a flame ionization detector (FID). A typical gas chromatogram of the reaction mixture after irradiation confirms the selective conversion of acetylene to ethylene without detectable over‐hydrogenation (Figures S6,S7). Systematic optimization of the system (Figures S8–S10) identified 100 µM catalyst and 250 µM photosensitizer in a H_2_O:ACN (1:1 *v*/*v*) with 10% *v/v* of TEA as optimal conditions. Under these conditions, the photocatalytic mixture produces 12.5 µmol of C_2_H_4_ after 4 h of irradiation with 99.9% selectivity (Figure 2a). Control experiments confirmed the photocatalytic nature of the reaction, as no ethylene formation was observed in the absence of catalyst, photosensitizer, sacrificial donor, or under dark conditions. In addition, screening of sacrificial donors demonstrated that TEA is the most effective under the working conditions (Figure S11).

At lower catalyst concentration (1 µM), the system achieves a turnover number (TON) of 670 after 48 h of irradiation, while maintaining a selectivity for ethylene over ethane of 99.6% (Figure 2b). This corresponds to a two‐fold improvement over a previously reported cobalt catalyst that does not employ Ru‐ or Ir‐based photosensitizer [12]. Notably, no other gaseous products were detected. To investigate the reason for saturation of ethylene production, re‐addition experiments were performed. Selective re‐addition of either CuPS and [Co(dmgH)_2_(Im)Cl] after 48 h, followed by further illumination, led to partial recovery of catalytic activity (Figure S12), pointing to a simultaneous depletion or degradation of both components.

To evaluate the performance under industrially relevant conditions, the CuPS/[Co(dmgH)_2_(Im)Cl] system was evaluated in the presence of excess C_2_H_4_ (1 vol.% C_2_H_2_, 30 vol.% C_2_H_4_, N_2_ balance). Under these competitive conditions, acetylene impurities were completely removed to undetectable levels (<5 ppm) within 105 min, while maintaining a selectivity of 99.3% toward ethylene (Figure 2c). This performance improves upon that reported by Hu et al., who achieved 99% acetylene conversion after 15 h under ethylene co‐feed conditions with a selectivity of 97.6% using Cu‐based photosensitizer [12].

The performance of the system containing CuPS was benchmarked against an analogous photocatalytic mixture employing the commercial tris(2,2′‐bipyridyl)ruthenium chloride ([Ru(bpy)_3_]^2+^) under comparable conditions, with irradiation wavelengths matching the absorption profile of the respective photosensitizers. After 4 h irradiation, the CuPS‐containing system produced approximately a three‐fold higher amount of C_2_H_4_ compared to the one containing [Ru(bpy)_3_]^2+^ and exhibited higher selectivity (Figure 2d). Electrochemical analysis revealed that CuPS has a more negative reduction potential in acetonitrile (E(CuPS/CuPS^•−^) = –1.92 V vs. Fc/Fc^+^) compared to [Ru(bpy)_3_]^2+^ (E(RuPS/RuPS^•−^) = −1.50 V vs. Fc/Fc^+^), rationalizing the enhanced performance of the copper‐based photosensitizer (Figure S13). Additionally, other commercial photosensitizers were also evaluated under the same working conditions and the CuPS‐containing system consistently exhibited the best performance among all the systems tested (Figure S14).

Finally, replacing the imidazole axial ligand of the cobaloxime with the commercially available pyridine analogue resulted in decreased activity, yielding 9.2 µmol of C_2_H_4_ after 4 h (Figure S15). Such behavior can be rationalized based on the basicity of the aromatic axial ligands, which influences their electron‐donating ability upon binding to cobalt and consequently the catalytic activity of the cobalt center [29]. Importantly, this comparison underscores how catalyst design translates into performance gains, while simultaneously highlighting the intrinsic tunability of the cobaloxime platform as a powerful handle for optimizing photocatalytic acetylene semi‐hydrogenation.

The photocatalytic activity of this system is not limited to the semi‐hydrogenation of acetylene. Upon illumination, the CuPS/[Co(dmgH)_2_(Im)Cl] system in the presence of phenylacetylene or 2‐methyl‐3‐butyn‐2‐ol produced styrene or 2‐methyl‐3‐buten‐2‐ol, respectively (Figures S16 and S17). These results demonstrate that this system can be extended to the semi‐hydrogenation of a broader range of alkynes, enabling the production of valuable intermediates widely used in industrial and pharmaceutical industries [34].

**FIGURE 3:**
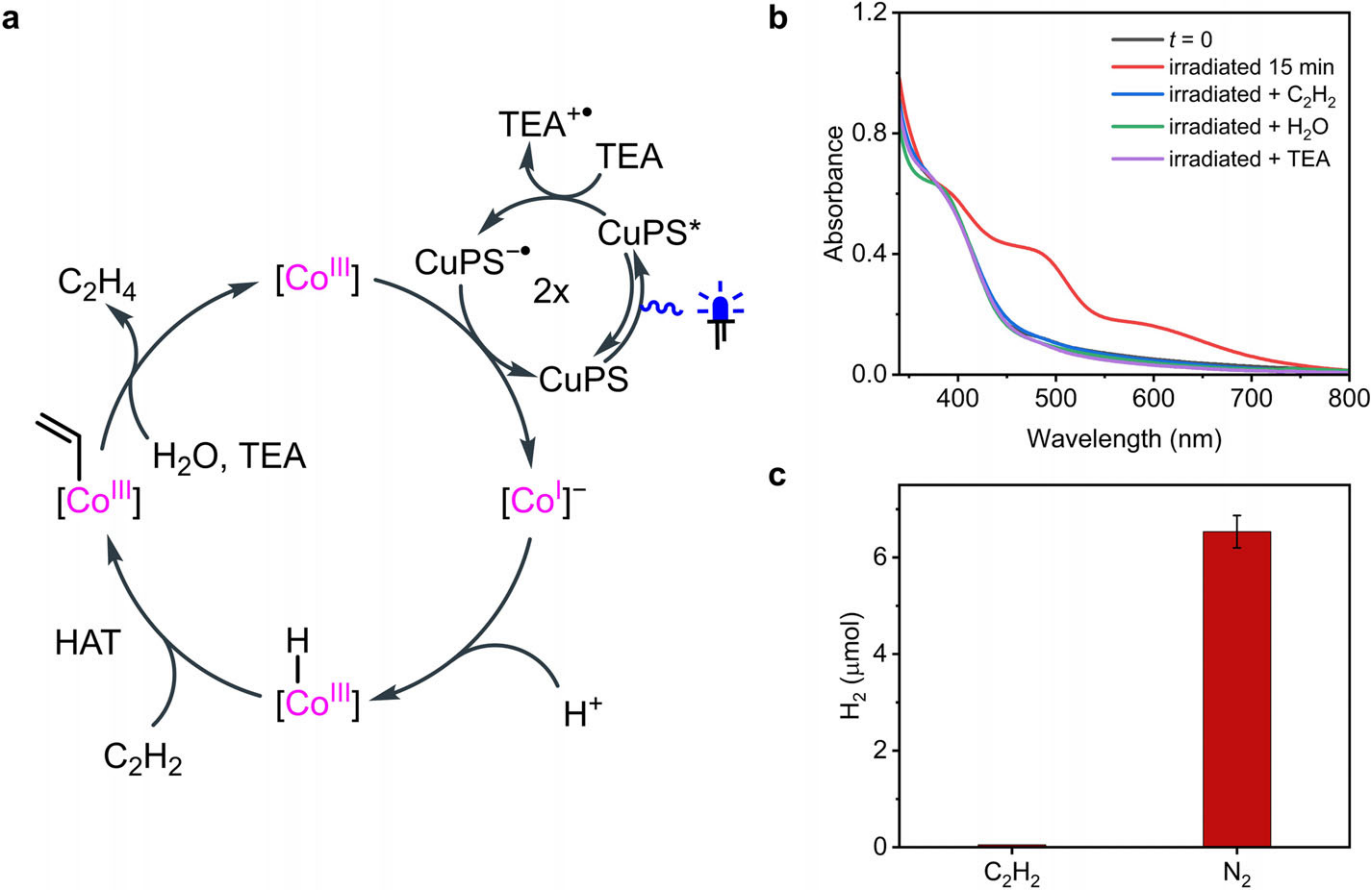
(a) Proposed catalytic mechanism for the photocatalytic reduction of C_2_H_2_ to C_2_H_4_ using [Co(dmgH)_2_(Im)Cl] and CuPS in H_2_O:ACN mixture (1:1 *v/v*) with 10% *v/v* TEA (pH = 11.0 ± 0.3). (b) UV–vis spectra of the system containing 100 µM [Co(dmgH)_2_(Im)Cl], 50 mM TEA, and 50 µM CuPS in ACN under various conditions (1.0 M H_2_O, ≥99.5 vol.% C_2_H_2_, or 0.7 M TEA). (c) H_2_ evolution by the CuPS/[Co(dmgH)_2_(Im)Cl] system under C_2_H_2_ or N_2_ in H_2_O:ACN mixture (1:1 *v/v*) with 10% *v/v* TEA (pH = 11.0 ± 0.3) irradiated (405 nm, 140 mW×cm^−2^) for 4 h.

Based on literature and experimental results, we propose a mechanism for the photocatalytic transfer hydrogenation of C_2_H_2_ mediated by the CuPS/[Co(dmgH)_2_(Im)Cl] system (Figure 3a). We first investigated the origin of the protons involved in catalysis. The removal of water from the reaction mixture results in an approximately 50% decrease in ethylene production (Figure S18), indicating the contribution of additional proton donors under catalytic conditions. Isotope‐labelling experiments provide further insight. When the reaction is performed in H_2_O, gas chromatography mass spectrometry (GC‐MS) analysis confirms ethylene (*m/z* = 28) as the sole product. In contrast, substitution with D_2_O leads to a partial isotopic scrambling: while isotope exchange generates C_2_D_2_ as the effective substrate, the reduced products exhibit dominant signals at *m/z* = 30 and 28, corresponding to partially and non‐deuterated ethylene species (Figure S19). These observations further indicate the involvement of proton sources beyond water. A plausible contributor is TEA, which is widely employed as a sacrificial donor and is known to reductively quench the excited state of photosensitizers, generating the redox‐non‐innocent transient radical TEA^•^ that can take part in an additional electron transfer [35]. Taken together, these observations support a mechanism in which both water and TEA‐derived species contribute to the addition of protons to make the ethylene product, while the excited state of photosensitizer (CuPS*) undergoes a reductive quenching pathway.

In our previously reported [Ru(bpy)_3_]^2+^‐sensitized cobaloxime system [11], the sacrificial donor BIH chemically reduces Co^III^ to Co^II^, which is subsequently reduced to the low valent Co^I^ species by the photogenerated [Ru(bpy)_2_(bpy)^•−^]^+^. In the present CuPS/[Co(dmgH)_2_(Im)Cl] system, however, TEA does not chemically reduce Co^III^, as evidenced by the unchanged UV–vis spectrum of [Co(dmgH)_2_(Im)Cl] upon addition of TEA (Figure S20). In particular, the appearance of the characteristic Co^II^ absorption band at 423 nm is not observed upon addition of TEA [36, 37, 38]. These findings indicate that the reduction of the cobalt center is mediated by photogenerated CuPS^•−^ species. We found direct evidence for the formation of Co^I^ intermediate through UV–vis spectroscopy. Upon irradiation of a solution containing [Co(dmgH)_2_(Im)Cl], CuPS, and TEA, two new absorption bands at 485 nm and 600 nm (Figure 3b) rapidly emerge, which are characteristic of Co^I^ species [36, 39]. Upon (i) purging with C_2_H_2_, (ii) addition of water, or (iii) addition of an excess of TEA, the characteristic Co^I^ absorption bands disappear and the initial profile is restored. These results indicate the formation of Co^I^ species and suggest that this active Co^I^ intermediate readily reacts with either C_2_H_2_ or a proton source under catalytic conditions. Acetylene reduction mediated by Co‐based catalysts is generally proposed to proceed via π‐complex formation or through a Co^III^─H intermediate [40]. The first route is generally less efficient, as its rate‐determining step involves the protonation of the π‐complex, whereas the latter involves reduction to a Co^I^ species that can readily engage protons, giving rise to active Co^III^─H. The relatively low bond dissociation of the Co‒hydride intermediate underpins both high activity and selectivity of these systems [41].

Consistent with this scenario, cobaloximes are well‐established molecular catalysts for H_2_ evolution, operating through Co^III^─H intermediates [22, 42, 43]. In agreement with this mechanism, H_2_ evolution is strongly suppressed under a C_2_H_2_ atmosphere compared to N_2_ (Figure 3c), indicating that hydrogen evolution and acetylene reduction compete for the same Co^III^─H intermediate, in accordance with our previous work [11].

Taken together, these results support a catalytic cycle in which photoexcited CuPS* is reductively quenched by TEA, generating CuPS^•–^ species that then reduce the cobalt center (Co^III^) twice to form Co^I^. Protonation of Co^I^ reacts with water or TEAH^+^ affords a reactive Co^III^─H intermediate. C_2_H_2_ then inserts into the Co^III^─H bond via a HAT pathway. An additional proton and electron are provided by H_2_O/TEA, and then the release of C_2_H_4_ regenerates Co^III^, closing the catalytic cycle.

## 3 Conclusions

To summarize, we have demonstrated that cobaloximes operating in tandem with a Cu‐based photosensitizer enable visible light‐powered C_2_H_2_ semi‐hydrogenation in a H_2_O:ACN mixture. The system operates under mild conditions, avoiding the use of high temperature and pressure, H_2_ gas feed, and noble metals, while delivering high activity and selectivity. The system remains efficient under an ethylene‐rich atmosphere, providing undetectable levels of C_2_H_2_ with an overall selectivity for ethylene of 99.3%. Cobaloximes are confirmed as excellent catalysts for C_2_H_2_ transfer hydrogenation due to their ability to undergo reduction to Co^I^ followed by protonation to form Co^III^─H. Importantly, comparative studies further show that fine‐tuning catalyst structure translates into catalytic efficiency, underscoring the intrinsic versatility of the cobaloxime scaffold for tuning reactivity and selectivity. Finally, the excellent performance achieved without relying on the benchmark [Ru(bpy)_3_]^2+^ photosensitizer highlights earth‐abundant copper photosensitizers as sustainable alternatives for photocatalytic purification processes relevant to industrial ethylene production.

Future developments should focus on moving to solvents like pure water that can also act as the proton donor for the reaction [8, 13, 18]. Overall, these findings lay the groundwork for further refinement of molecular photocatalytic systems for this relevant chemical transformation, their implementation in photoelectrochemical or continuous‐flow configurations, and their eventual application to other selective hydrogenation reactions of industrial relevance.

## Author Contributions

F.A. proposed and supervised the project. F.A., L.Ð., and A.F. designed the experiments. A.F. synthesized the cobaloxime, B.A. synthesized the CuPS. A.F. characterized the materials, with the help of M.B and B.A. A.F. and S.S. performed the photocatalytic experiments. A.F. and F.A. wrote the manuscript. All authors were involved in manuscript revision and have approved the final version of the manuscript.

## Conflicts of Interest

The authors declare no conflicts of interest.

## Supporting information




**Supporting File**: chem71119‐sup‐0001‐SuppMat.pdf.

## Data Availability

The data supporting this article have been included as part of the Supporting Information. Supporting Information: Figures S1–S20 and further experimental details. The authors have cited additional references within the Supporting Information (Ref. [8, 29, 33, 44]). See DOI: [URL – format https://doi.org/DOI
