# Supplementary material for: Visible‐Light Driven Acetylene Semi‐Hydrogenation to Ethylene With a Copper Photosensitizer and a Cobaloxime Catalyst
Source: Chemistry. 2026 May 16;32(33):e71119. doi: 10.1002/chem.71119 (PMC13532131; doi:10.1002/chem.71119)
Supplement: Supplementary file 1 [file CHEM-32-e71119-s001.pdf]

Supplementary Information for

Visible-Light Driven Acetylene Semi-Hydrogenation to Ethylene with a Copper Photosensitizer and a Cobaloxime Catalyst

Anna Fortunato*, Sharon Silloni, Brando Adranno, Marianna Barbieri, Luka Đorđević, and Francesca Arcudi*

Department of Chemical Sciences, Università di Padova, Via Marzolo 1, Padova, Italy

*Corresponding authors. Email: anna.fortunato@unipd.it, francesca.arcudi@unipd.it

Table of Contents

Materials and Methods 3

Safety Warning 3

Materials 3

Synthesis of ([Co(dmgH)$_2$(Im)Cl] 3

Synthesis of CuPS 4

Photocatalytic Reactions 4

Chromatographic Detection of Gases 5

Chromatographic Detection of Liquid 7

Absorption Spectroscopy 7

Photoluminescence Spectroscopy 7

^{1}H NMR 7

Electrochemical characterization 7

Mass Spectrometry 7

Supplementary Figures 8

Supplementary References 28

Materials and Methods

Safety Warning

Acetylene is an extremely flammable gas. Buildup of acetylene vapors can result in fire or explosions if triggered by sparks. Acetylene may displace oxygen and cause rapid suffocation. In our experimental setup, the acetylene cylinder was fitted with a CGA 510 regulator equipped with a flashback arrestor and connected, through stainless steel tubing and a flow regulator, to a purging station that was placed inside a fume hood.[1] A Snoop® solution was applied to fittings and joints to inspect for leaks, until no bubble formation was observed. There was no electrical equipment in the fume hood.

Materials

Cobalt chloride hexahydrate ($CoCl_2 \cdot 6H_2O$, Merck, 98%), dimethylglyoxime (Sigma-Aldrich, ≥99%), imidazole (Sigma-Aldrich, 99%), potassium hydroxide (KOH, Sigma-Aldrich, flakes, 90%) triethylamine (TEA, Sigma-Aldrich, ≥99.0%), tris(2,2′-bipyridyl)dichlororuthenium(II) hexahydrate ($[Ru(bpy)_3]^{2+}$, Sigma-Aldrich, 99.95%), chloro(pyridine)bis(dimethylglyoximato)cobalt(III) ($[Co(dmgH)_2pyCl]$, Sigma-Aldrich, 3416305G), tetrakis(acetonitrile)copper(I) hexafluorophosphate ($[Cu(MeCN)_4]PF_6$, Sigma-Aldrich, 97%), 4,5-bis(diphenylphosphino)-9,9-dimethylxanthene (xantphos, TCI, >98.0%), bathocuproine (TCI, >95.0%) ethanol (EtOH, Sigma-Aldrich), rose bengal (Sigma-Aldrich, ≥96%), 1,2,3,5-tetrakis(carbazol-9-yl)-4,6-dicyanobenzene (4CzIPN, BLDPharm, 97%), eosin Y (Sigma-Aldrich, 99%), 9-mesityl-3,6-di-*tert*-butyl-10-phenylacridinium tetrafluoroborate (Sigma-Aldrich, ≥95%), ascorbic acid (HAsc, Sigma-Aldrich, ≥99.0%), triethanolamine (TEOA, Sigma-Aldrich, ≥99.0%), methanol (MeOH, Sigma-Aldrich, ≥99%), and D_2O (Sigma-Aldric, 99.9% atom D) and were used as received.

Ultrapure water (MilliQ, >18.3 MΩ cm) was obtained using a PURELAB® flex 4system.

Synthesis of ($[Co(dmgH)_2(Im)Cl]$

Chloro(imidazole)cobaloxime $[Co(dmgH)_2(Im)Cl]$ was synthesized according to a modified literature procedure.[2] To a solution of $CoCl_2 \cdot 6H_2O$ (300.0 mg, 1.26 mmol, 1.0 equiv.), in absolute EtOH (13 mL) at room temperature, was added dimethylglyoxime (292.0 mg, 2.52 mmol, 2.0 equiv.) followed by KOH (70.0 mg, 1.26 mmol, 1.0 equiv.). The solution was warmed to 80 °C for 1 h, then imidazole (85.8 mg, 1.26 mmol, 1.0 equiv.) was added to the solution, which was then allowed to cool to r.t.. A stream of compressed air was passed through to the mixture for 1 h via a Pasteur pipette.

The product was collected by centrifugation, rinsed with water (15 mL), EtOH (2 × 15 mL), and Et_2O (3 × 15 mL) and then dried under vacuum overnight to give [Co(dmgH)$_2$(Im)Cl] as a brown powder (450.0 mg, 90% yield). ^{1}H NMR (300 MHz, DMSO-d_6): δ = 18.67 (*s*, 2H, OH), 12.78 (*s*, 1H), 7.28 (*s*, 1H), 7.08 (*s*, 1H), 6.43 (*s*, 1H), 2.30 (*s*, 12H). *Anal*. Calc. for $C_{11}H_{18}ClCoN_6O_4$: C, 33.65; H, 4.62; N, 21.40. Found: C, 33.57; H, 4.80; N, 21.20.

Synthesis of CuPS

CuPS was synthesized according to a modified one-pot literature procedure.[3] [Cu(MeCN)$_4$]PF_6 (200.0 mg, 0.54 mmol, 1.0 eq.) and xantphos (310.5 mg, 0.54 mmol, 1.0 eq.) were placed in a dried Schlenk tube under N_2 atmosphere and were dissolved in 10.8 mL of anhydrous dichloromethane. The mixture was let stirring in reflux overnight under N_2 atmosphere to obtain a light-yellow solution. After cooling to room temperature, a solution of bathocuproine (193.4 mg, 0.54 mmol, 1.0 eq.) in 2.9 ml of dichloromethane was added to the Schlenk tube, immediately turning the solution to dark orange/red color. The solution was then let stir in reflux for 2 hours. The reaction was let to cool down to room temperature before adding 100 mL of *n*-hexane to obtain the precipitation of a red sticky solid, which slowly turned to an orange powder with stirring and further addition of 450 mL of *n*-hexane. The product was collected by filtration, washed with *n*-hexane and dried under vacuum as a dark yellow/orange powder (497.6 mg, 81% yield). ^{1}H NMR (400 MHz, DMSO-d_6) δ = 7.85 (*dd*, J = 7.8, 1.4 Hz, 2H), 7.72 (*d*, J = 10.5 Hz, 4H), 7.66 – 7.53 (*m*, 10H), 7.32 (*t*, J = 7.5 Hz, 6H), 7.17 – 7.03 (*m*, 16H), 7.02 – 6.97 (*m*, 2H), 2.32 (*s*, 6H), 1.72 (*s*, 6H). ESI TOF *m/z* (positive mode) 1001.2977 (calc. *m/z* = 1001.2851).

Photocatalytic Reactions

Samples were prepared in an 8.0 mL screw cap vial (548-0821A, VWR) equipped with a micro stir bar (10 mm, VWR) and sealed with silicone/PTFE septum (548-0487A, VWR) and cap (open top, 548-2416A, VWR).

Vials for the semi-hydrogenation of acetylene were sealed and purged, through a flow regulator (at 130 mm using a Key Instruments 150 mm glass tube flowmeter), either for (*i*) 10 minutes with He (or Ar) followed by 5 minutes with C_2H_2 (≥99.5 vol.%, Airgas) or (*ii*) for 15 minutes with C_2H_4/C_2H_2 mixture (1 vol.% C_2H_2, 30 vol.% C_2H_4, N_2 balance, Airgas) or (*iii*) 15 minutes with Ar by using steel needles inserted through the septum as inlet (inserted into the solution) and outlet (venting the headspace to the surrounding atmosphere). After purging for the time specified, the pressure of the headspace was then equilibrated to 1 atm. The vials containing CuPS photosensitizer were then illuminated using a homebuilt photoreactor made of 405 nm (U60 (400–410 nm)) LEDs (High Power LED Star, LEDsupply.com) with a light intensity of 140 mW×cm^{-2} (measured using an Optical Power

Meter PM100D with Optical Sensor S120VC from Thorlabs). For the screening of different photosensitizers, LEDs of appropriate emission wavelengths were used in homebuilt photoreactors. Specifically, vials containing $[Ru(bpy)_3]^{2+}$ or 9-mesityl-3,6-di-*tert*-butyl-10-phenylacridinium tetrafluoroborate were illuminated with royal blue (450 nm) LEDs (Cree XLamp XP-E2 Color High Power LED Star, LEDsupply.com); vials containing Eosin Y were illuminated using green (520 – 535 nm) LEDs (Cree XLamp XP-E2 Color High Power LED Star, LEDsupply.com); vials containing Rose Bengal PS were illuminated using PC Amber (565 – 595 nm) LEDs (Cree XLamp XP-E2 Color High Power LED Star, LEDsupply.com); vials containing 4CzIPN PS were illuminated using a U70 (410 – 420 nm) LEDs (Ultraviolet (UV−A) High Power LED Star, LEDsupply.com). In all experiments, the light intensity was maintained at 140 $mW \times cm^{-2}$ (measured using an Optical Power Meter PM100D with Optical Sensor S120VC from Thorlabs). Each vial was suspended on top of a single LED, equipped with a lens, using a homebuilt sample holder. The vials were continuously stirred at 500 rpm during irradiation. To prevent the system from overheating, two fans were employed, one positioned beneath the photoreactor and a second placed adjacent to the setup, directed toward the electrical components.

Vials for the semi-hydrogenation of alkynes (i.e. phenylacetylene and 2-methyl-3-buten-2-ol) were charged with $[Co(dmgH)_2(Im)Cl]$ (10 mol%), CuPS (250 μM), and alkyne (10 mM) in H_2O:ACN (1:1 *v*/*v*) with 10% *v*/*v* of TEA. Then the vials were sealed and purged, through a flow regulator (at 130 mm) for 15 minutes with Ar by using steel needles inserted through the septum as inlet (inserted into the solution) and outlet (venting the headspace to the surrounding atmosphere). The vials were then illuminated using a homebuilt photoreactor made of 405 nm (U60 (400–410 nm)) LEDs (High Power LED Star, LEDsupply.com) with a light intensity of 140 $mW \times cm^{-2}$ (measured using an Optical Power Meter PM100D with Optical Sensor S120VC from Thorlabs). Upon irradiation, the photocatalytic mixtures were extracted with hexane and then analyzed by GC.

Chromatographic Detection of Gases

GC-MS experiments were performed on an Agilent Technologies 7890A GC system coupled with a 5975C VL MSD with Triple-Axis Detector. The GC was equipped with a HPPLOT Q column, the inlet temperature was 200 °C, the He carrier gas flow was 1 $mL \times min^{-1}$ at a pressure of 4.1 psi. For the detection of ethylene, acetylene, and ethane the oven temperature was kept at 110 °C for 6.5 min, and then heated to 250 °C using a 15 $°C \times min^{-1}$ ramp (total run time 18.8 min). Headspace samples were manually injected using gas-tight Hamilton syringes (100 μL).

For the detection and quantification of acetylene and ethane in the photoreduction, GC experiments were performed on an Agilent Technologies 8860 GC system coupled with flame ionization detector (FID) and a thermal conductivity (TCD) detector. The system was equipped with a HP-PLOT U and

a MS-5A column, Ar carrier gas flow was 6 mL×min^{-1} at a constant pressure of 11.121 psi, the FID and TCD detectors were kept at 250 °C. For the detection of ethylene, acetylene, and ethane the oven temperature was kept at 40 °C for 5 min, then heated to 110 °C using a 20 °C×min^{-1} ramp, and kept at 110 °C for 9 min (total run time 17.5 min). Headspace samples were injected using PAL3 series 2 Autosampler Systems equipped with a gas-tight syringe (SGE autosampler syringe) injecting 100 µL. Calibration curves for C_2H_4, C_2H_2, C_2H_6 and H_2 were collected by injecting known quantities of gas mixture standards containing C_2H_4 (99.95 vol% or 2.5 vol% or 30 vol%), C_2H_2 (2.5 vol.% or 1 vol.%), C_2H_6 (2.5 vol.%), or H_2 (99.95 vol.% or 2.5 vol.%). All calibration curves are reported in Figure S6. Injections were performed either using gas-tight Hamilton syringes (10 − 100 µL) or using PAL3 series 2 Autosampler Systems equipped with a gas-tight syringe (SGE autosampler syringe) injecting 100 µL. Calibration injections were performed at least in triplicate.

The selectivity for C_2H_4 is reported as >99.9% when no quantifiable C_2H_6 and H_2 are detected in the gas chromatograms. Turnover number (TON) of C_2H_4 produced and selectivity for ethylene vs. C_2H_6 and H_2 ($S_{C_2H_4}^{tot}$) and vs. just C_2H_6 ($S_{C_2H_4}^{C_2H_6}$) for the photoreduction of C_2H_2 (≥99.5 vol.%) were calculated as follows:

$$\mathrm{TON_{C_2H_4}} = \frac{\mathrm{mol_{C_2H_4}}}{\mathrm{mol_{[Co(dmgH)_2(Im)Cl]}}}$$

$$S_{C_2H_4}^{tot}\ (\%) = \frac{\mathrm{mol_{C_2H_4}}}{\mathrm{mol_{C_2H_4}} + \mathrm{mol_{H_2}} + \mathrm{mol_{C_2H_6}}} \times 100$$

$$S_{C_2H_4}^{C_2H_6}\ (\%) = \frac{\mathrm{mol_{C_2H_4}}}{\mathrm{mol_{C_2H_4}} + \mathrm{mol_{C_2H_6}}} \times 100$$

The quantification of residual acetylene ($\mathrm{C_{C_2H_2}}$) in the photoreduction of the C_2H_4/C_2H_2 mixture (1 vol.% C_2H_2, 30 vol.% C_2H_4, He balance, Airgas) and the selectivity for ethylene vs C_2H_2 and C_2H_6 were calculated as follows:

$$\mathrm{C_{C_2H_2}}(\%) = \frac{[\mathrm{C_2H_2}]_{\mathrm{feed}} - [\mathrm{C_2H_2}]_{\mathrm{x}}}{[\mathrm{C_2H_2}]_{\mathrm{feed}}} \times 100$$

$$\mathrm{S_{C_2H_4}}(\%) = \frac{[\mathrm{C_2H_2}]_{\mathrm{feed}} - [\mathrm{C_2H_2}]_{\mathrm{x}}}{[\mathrm{C_2H_2}]_{\mathrm{feed}} - [\mathrm{C_2H_2}]_{\mathrm{x}} - [\mathrm{C_2H_6}]}$$

where $[C_2H_2]_{feed}$ represents the acetylene concentration in the feed (1.0×10^4 ppm) and $[C_2H_2]_x$ and $[C_2H_6]$ are the concentrations of acetylene and ethane in the product. The change in ethylene

concentration cannot be accurately measured because of the excess of ethylene in the gas feed and therefore is not used for calculating the selectivity.[4]

Chromatographic Detection of Liquid

For detection of styrene and 2-methyl-3-buten-2-ol, GC analyses were performed in a GC 8860. The GC was equipped with a ZB-WAXplus Phenomenex column (30 m, 0.25 id, 0.25 thickness), the inlet temperature was 250 °C, the He carrier gas flow was 1.2 mL×min^{-1}. The oven temperature was kept at 40 °C for 5 min, and then heated to 250 °C using a 15 °C×min^{-1} ramp, with solvent delay 2.5 min (total run time 23.5 min). The liquid samples were manually injected using Hamilton syringes (1 μL).

Absorption Spectroscopy

Absorption spectra of compounds were recorded at room temperature with an Agilent Cary 60 UV-Vis spectrophotometer, using quartz cells with a path length of 1.0 cm.

Photoluminescence Spectroscopy

Fluorescence emission spectra were recorded on an Agilent Cary Eclipse fluorescence spectrofluorometer using quartz cells with a path length of 1.0 cm.

^{1}H NMR

NMR spectra were obtained on a Varian 400 MHz NMR (400 MHz ^{1}H) spectrometer at room temperature (298 K). The resonance multiplicity is described as *s* (singlet), *d* (doublet), *dd* (doublet of doublets), *t* (triplet), *m* (multiplet), *bs* (broad singlet).

Electrochemical characterization

Cyclic voltammetry (CV) was performed on a Pine Research WaveDriver 100 potentiostat at room temperature, employing a standard three-electrode single-compartment cell: glassy carbon electrode (GCE, CH Instruments, d = 3 mm) as working electrode, a Pt wire as counter electrode, Ag wire as pseudo-reference electrode and ferrocene was used as internal reference. Working and reference electrodes were polished on a felt pad with 0.3 or 0.05 μm Al_2O_3 suspensions, sonicated in deionized water for about 30 seconds and washed/dried before each experiment; the Pt wire was polished with sandpaper. $TBAPF_6$ (0.1 M) was used as electrolyte and the cell environment was purged for 15 minutes with N_2 before recording the CV. A blank scan was recorded before each sample (scan rate = 100 mV×s^{-1}). The CV scans were initiated by sweeping toward negative potentials.

Mass Spectrometry

Mass spectrometry was performed at the Department of Pharmaceutical and Pharmacological Sciences (University of Padova) with a Xevo G2-S QTof. The sample was prepared by dissolving a small amount of the compound in ACN to obtain a 10^{-4} M solution.

Supplementary Figures

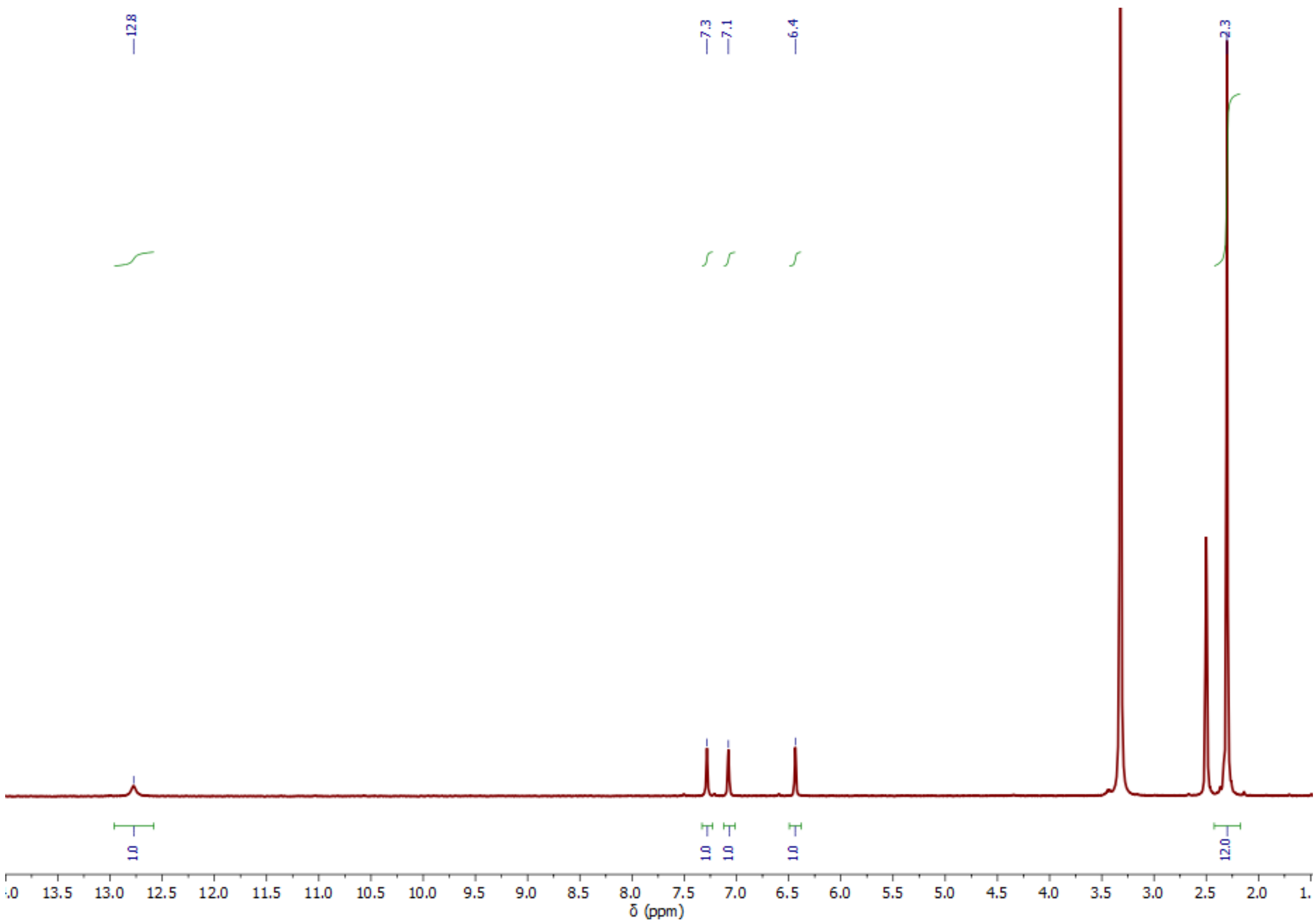

Figure S1. ^{1}H NMR (DMSO-d_6) spectrum of [Co(dmgH)$_2$(Im)Cl].

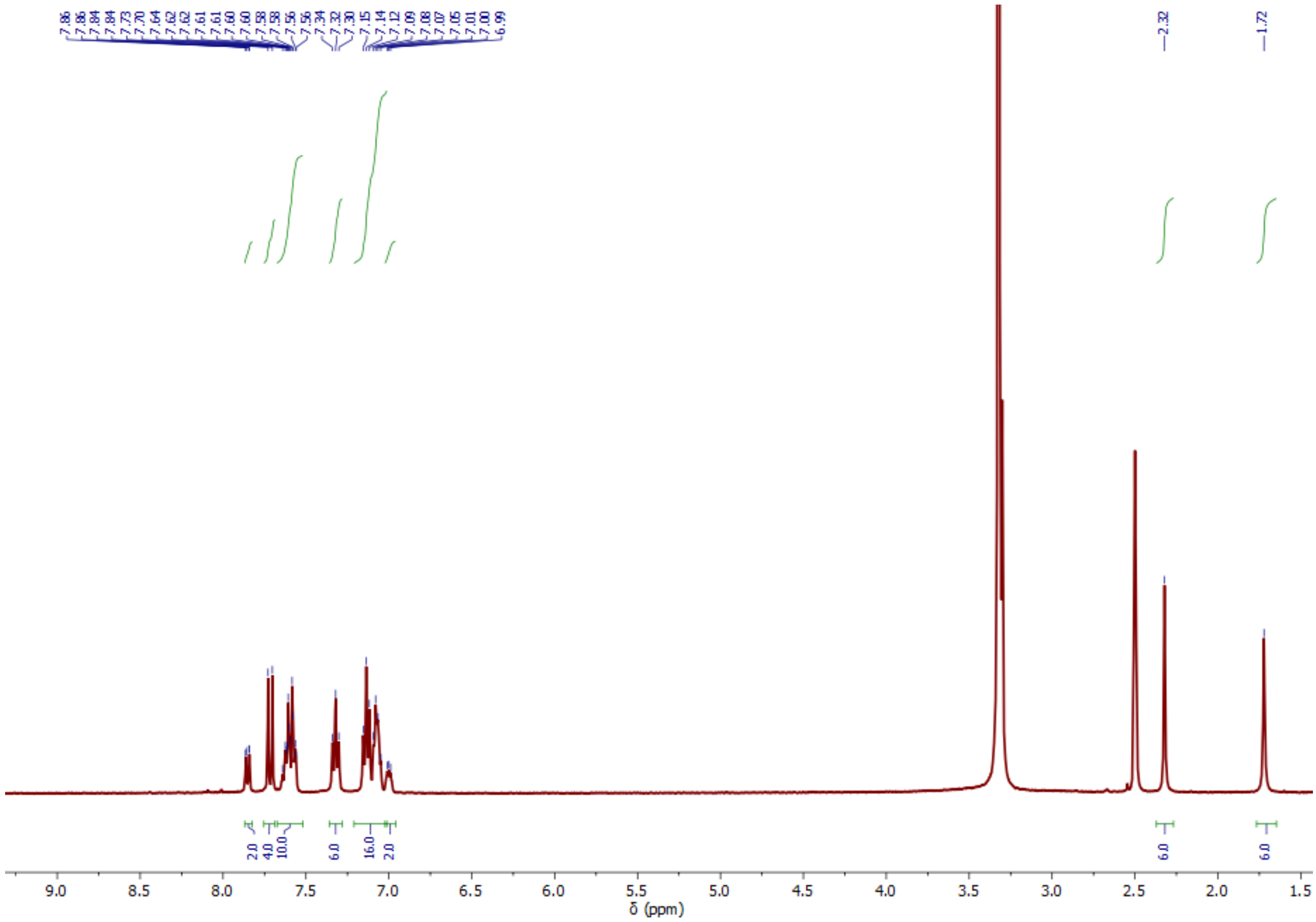

Figure S2. ^{1}H NMR (DMSO-d_6) spectrum of CuPS.

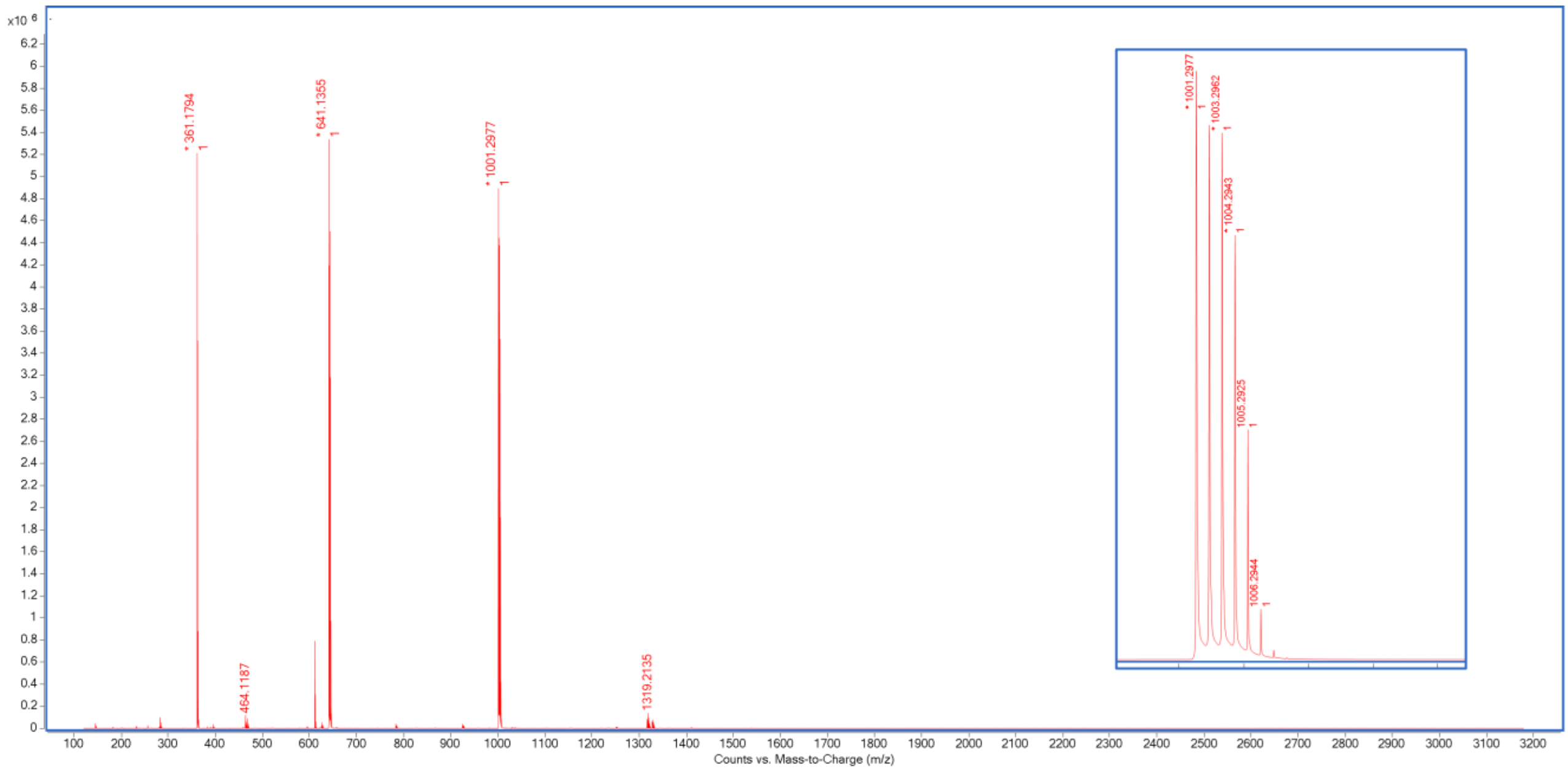

Figure S3. HRMS (ESI positive) for compound CuPS (*m/z* =1001.30, $[CuPS]^+$, insert). Fragment ions detected at *m/z* = 361.18 and 641.13 are attributed to the $[\text{Bathocuproine–H}]^+$ and $[\text{Xantphos–Cu}]^+$, respectively, formed upon fragmentation under positive ionization conditions.

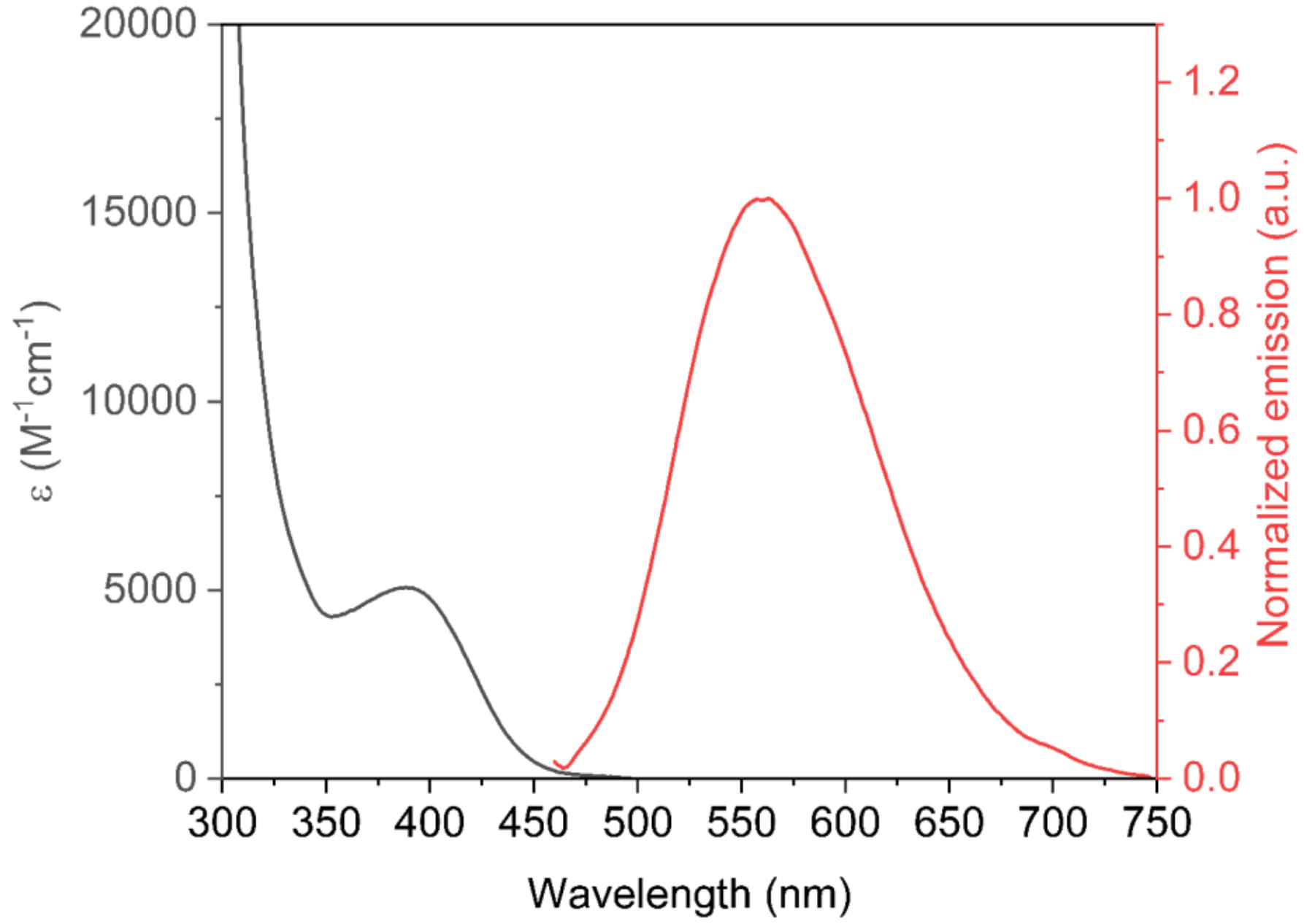

Figure S4. Molar absorption coefficient (gray) and normalized emission spectra of CuPS in ACN.

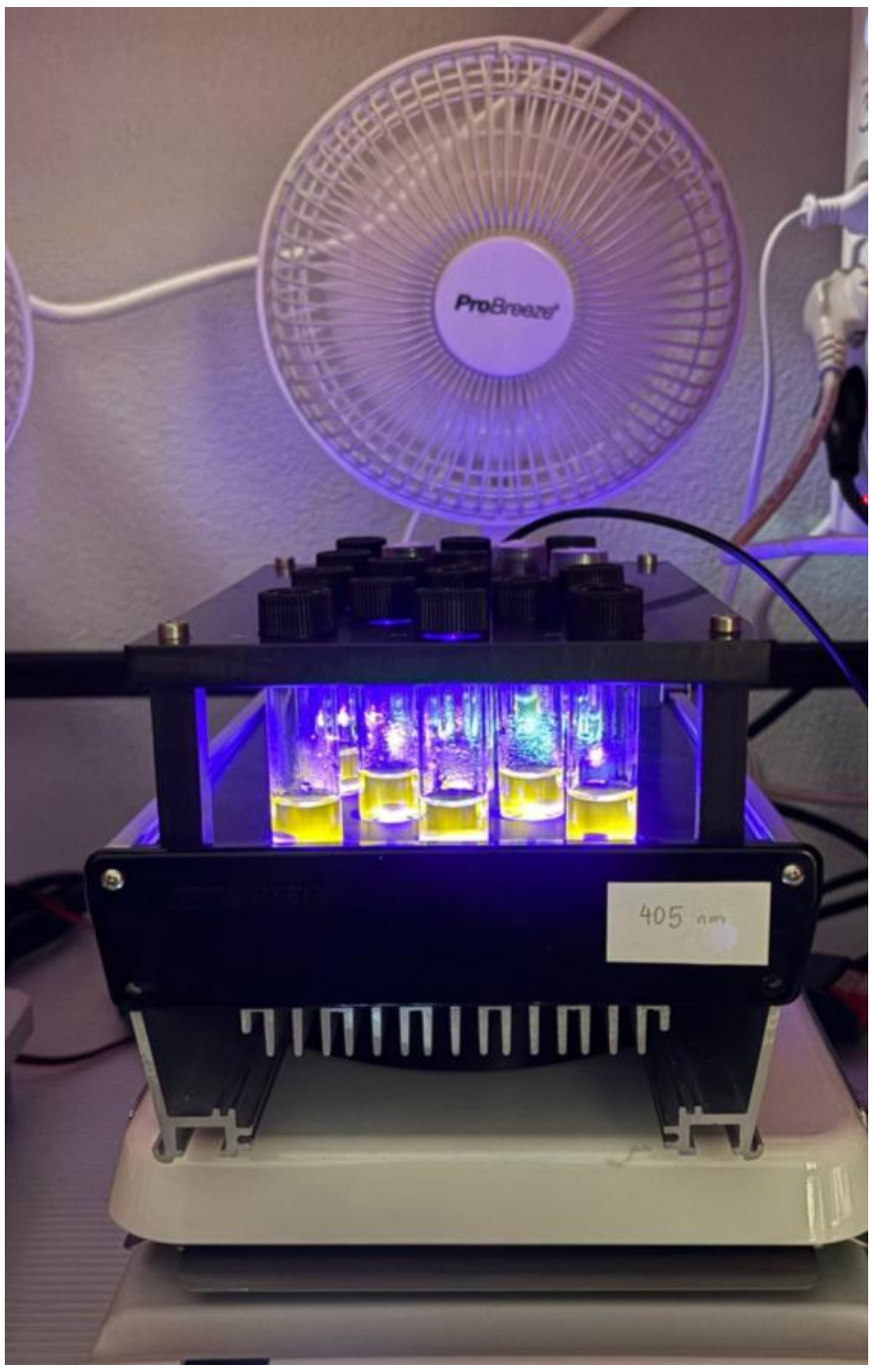

Figure S5. Photograph of the homebuilt photoreactor.

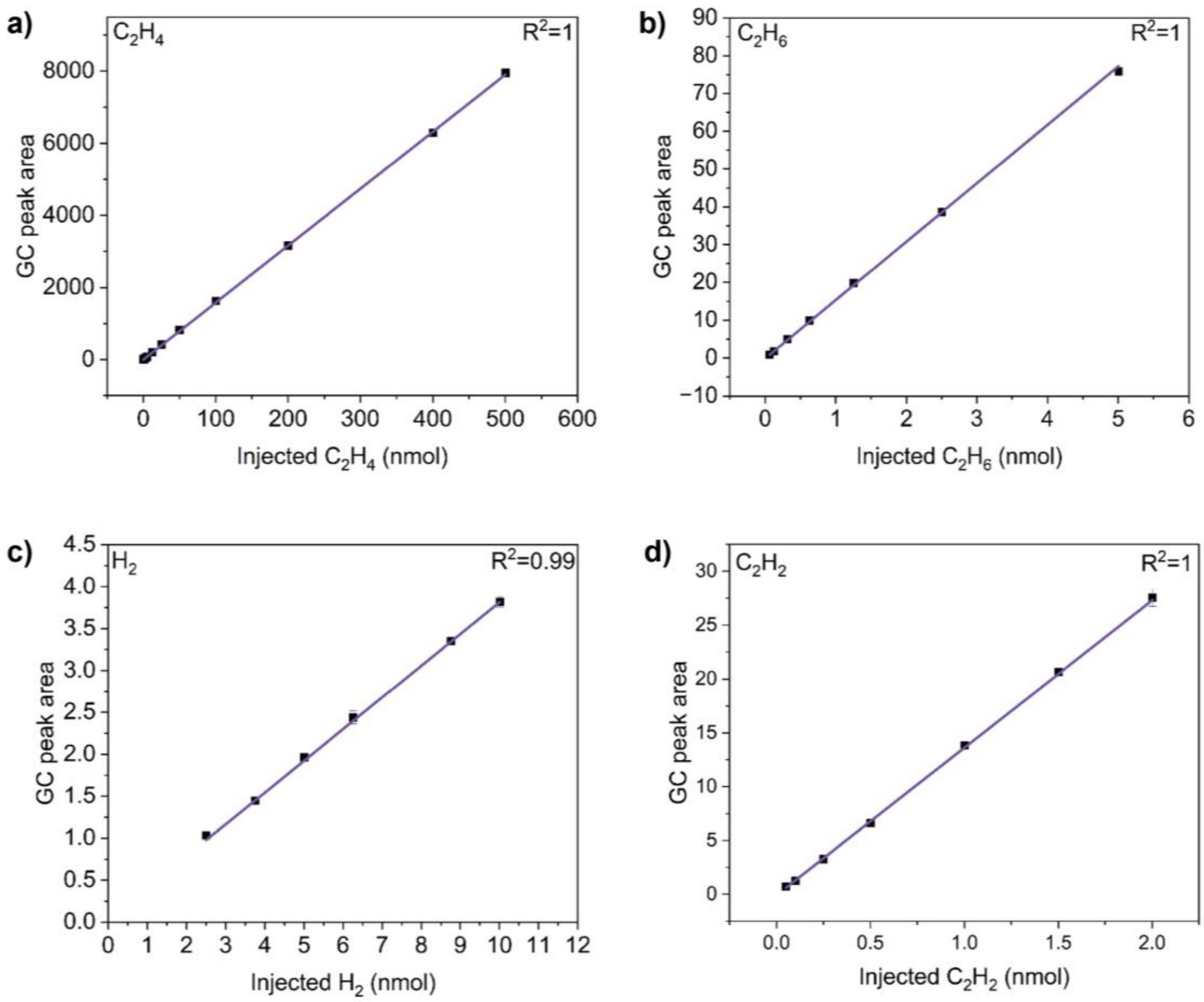

Figure S6. a) Calibration curve for C_2H_4 with the corresponding coefficient of linear correlation (R^2). b) Calibration curve for C_2H_6 with the corresponding coefficient of linear correlation (R^2). c) Calibration curve for H_2 with the corresponding coefficient of linear correlation (R^2). d) Calibration curve for C_2H_2 with the corresponding coefficient of linear correlation (R^2).

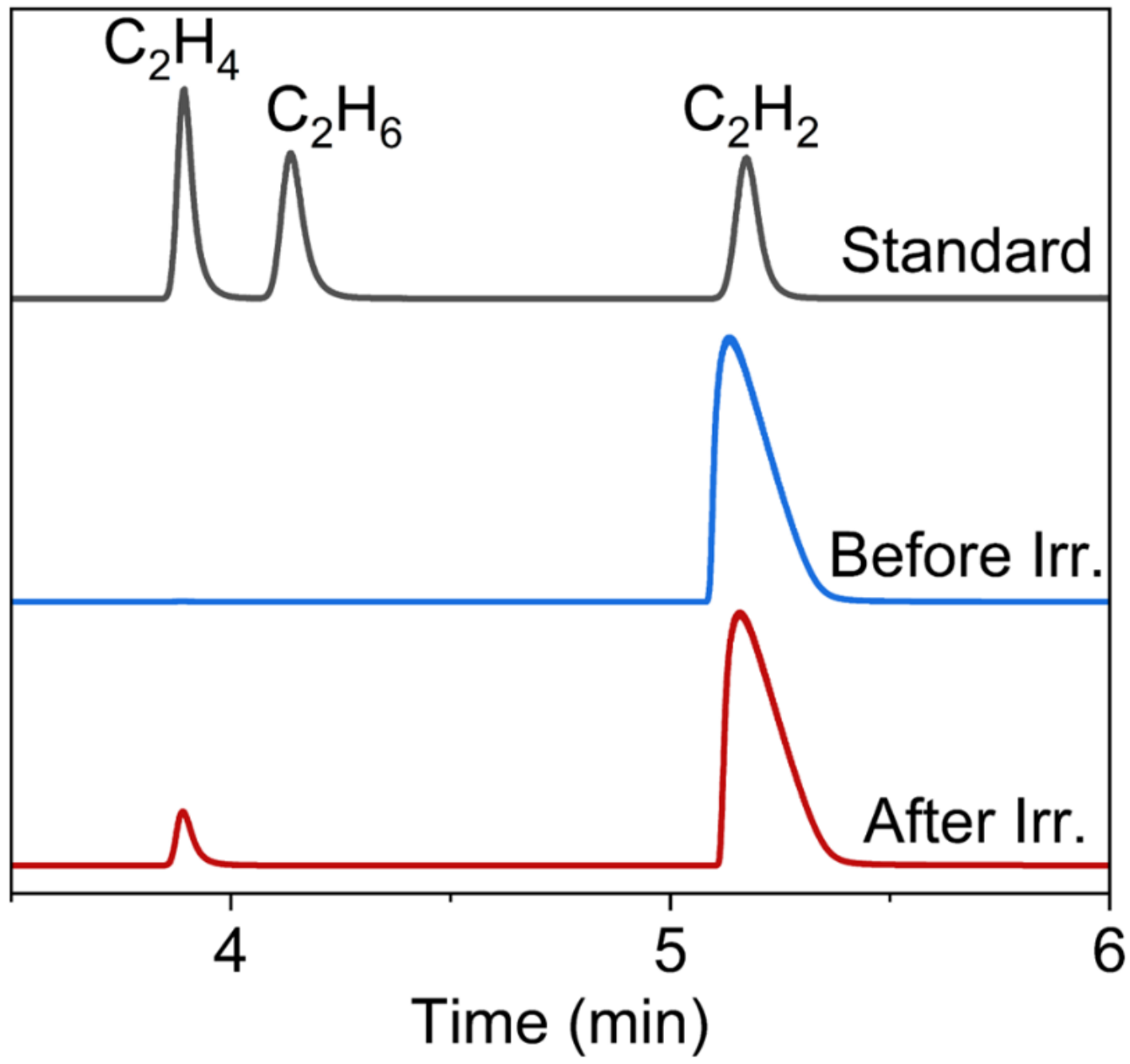

Figure S7. Typical gas chromatographs observed for the photoreduction of acetylene (retention time of C_2H_4, C_2H_6 and C_2H_2) detected with flame ion detection of a gas standard mixture (gray) containing C_2H_4 (2.5 vol.%), C_2H_6 (2.5 vol.%) and C_2H_2 (2.5 vol.%) and the optimized system under C_2H_2 (≥99.5 vol.%) before (blue) and after (red) irradiation (405 nm, 140 mW×cm^{-2}) for 4 h containing 100 µM [Co(dmgH)$_2$(Im)Cl] in H_2O:ACN (1:1 *v*/*v*) mixture with 10% *v*/*v* TEA (pH = 11.0 ± 0.3).

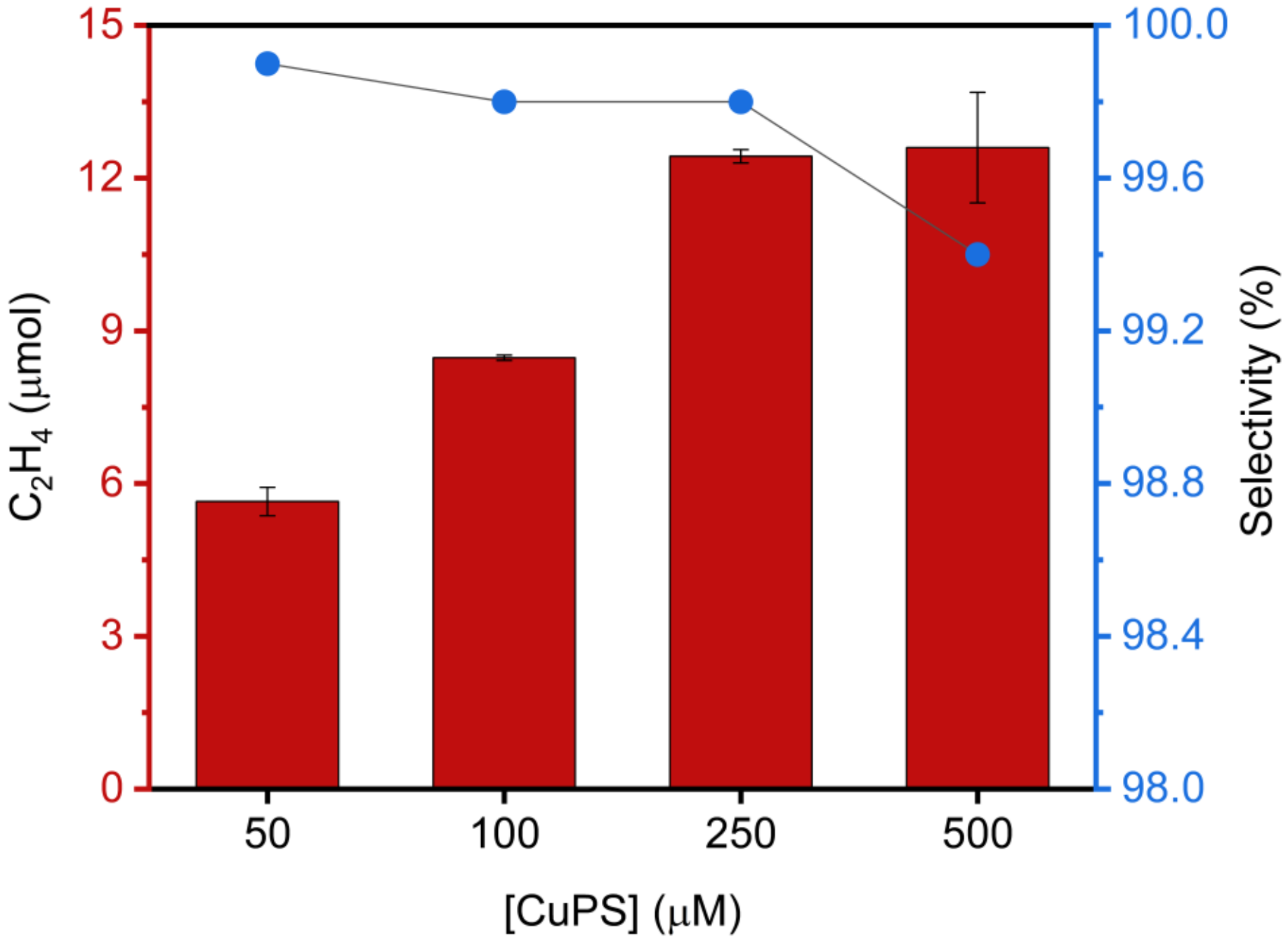

Figure S5. Amount of C_2H_4 (µmol) produced and selectivity (%) for C_2H_4 by the [Co(dmgH)$_2$(Im)Cl]/CuPS system containg 100 µM [Co(dmgH)$_2$(Im)Cl] in H_2O:ACN (1:1 *v*/*v*) mixture with 10% *v*/*v* TEA (pH = 11.0 ± 0.3) under C_2H_2 (≥99.5 vol.%) after irradiation with 405 nm light (140 mW×cm^{-2}) for 4 h through variation of [CuPS]. Error bars indicate standard error of the mean, calculated from three runs.

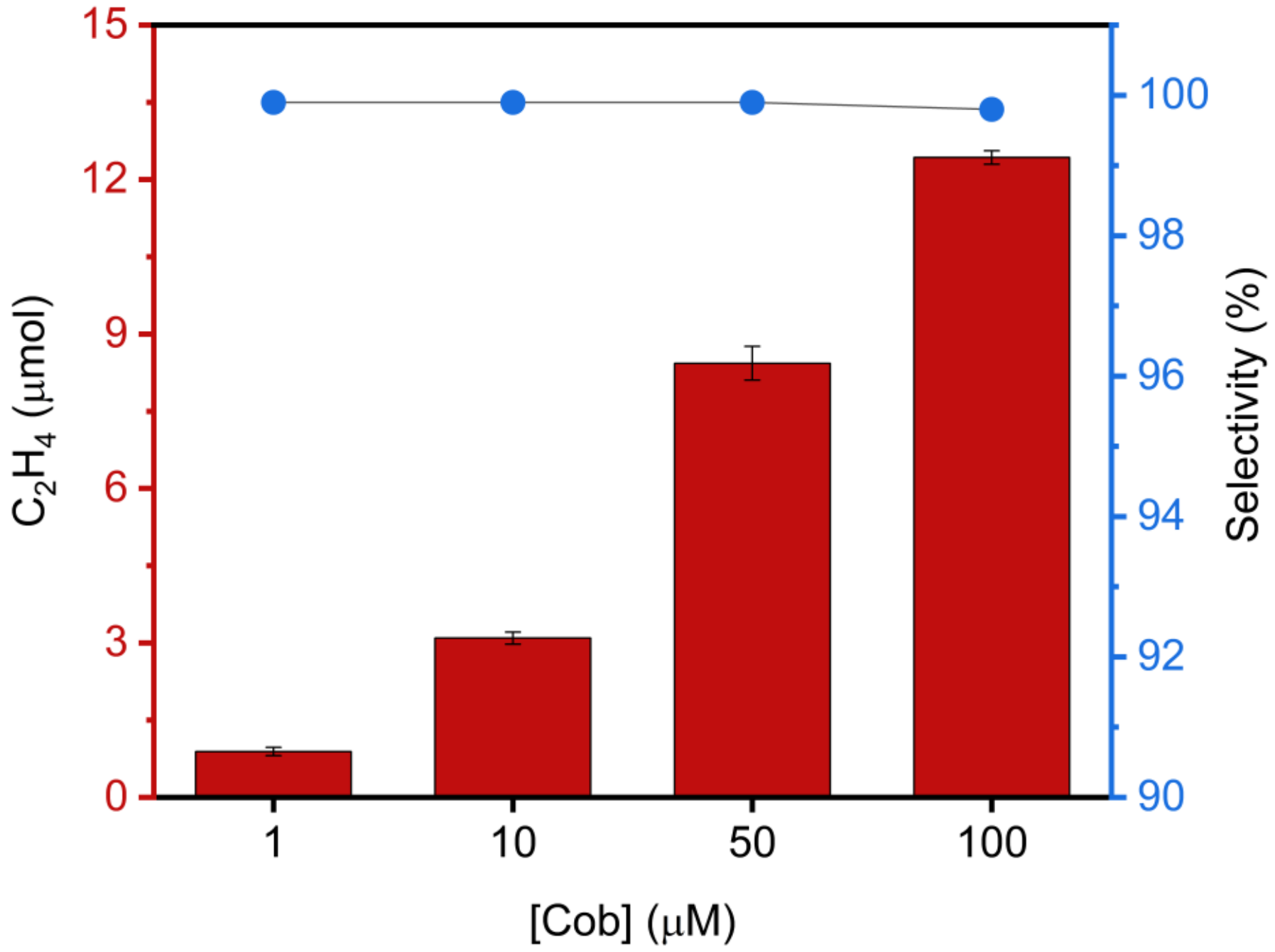

Figure S6. Amount of C_2H_4 (μmol) produced and selectivity (%) for C_2H_4 by the [Co(dmgH)$_2$(Im)Cl]/CuPS system containg 250 μM CuPS in H_2O:ACN (1:1 *v*/*v*) mixture with 10% *v*/*v* TEA (pH = 11.0 ± 0.3) under C_2H_2 (≥99.5 vol.%) after irradiation with 405 nm light (140 mW×cm^{-2}) for 4 h through variation of [Co(dmgH)$_2$(Im)Cl]. Error bars indicate standard error of the mean, calculated from three runs.

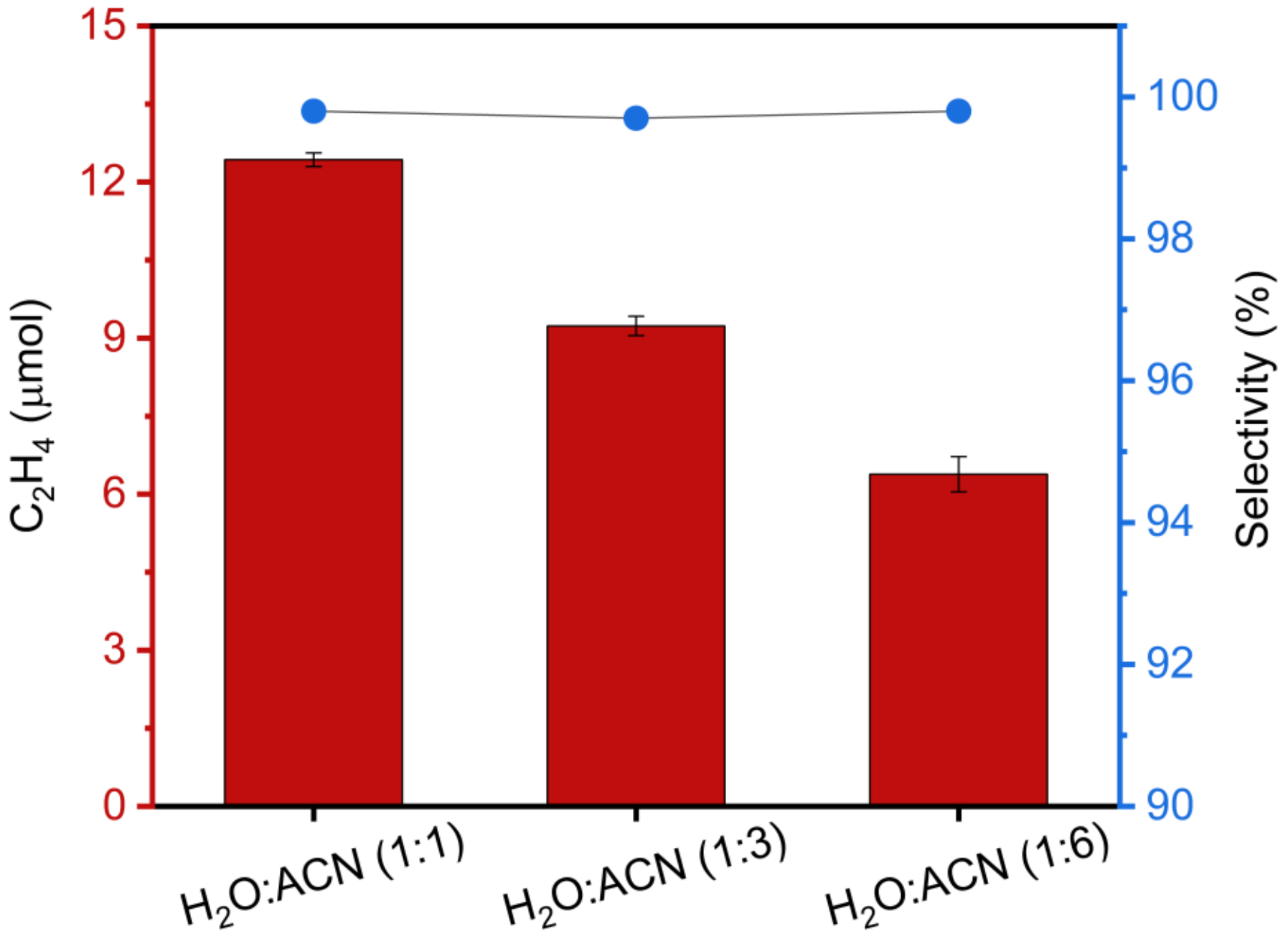

Figure S7. Amount of C_2H_4 (nmol) produced and selectivity (%) for C_2H_4 by the [Co(dmgH)$_2$(Im)Cl]/CuPS system containg 100 µM [Co(dmgH)$_2$(Im)Cl] and 250 µM CuPS in H_2O:ACN mixture with 10% *v*/*v* TEA (pH = 11.0 ± 0.3) under C_2H_2 (≥99.5 vol.%) after irradiation with 405 nm light (140 mW×cm^{-2}) for 4 h through variation of H_2O:ACN ratio. Error bars indicate standard error of the mean, calculated from three runs.

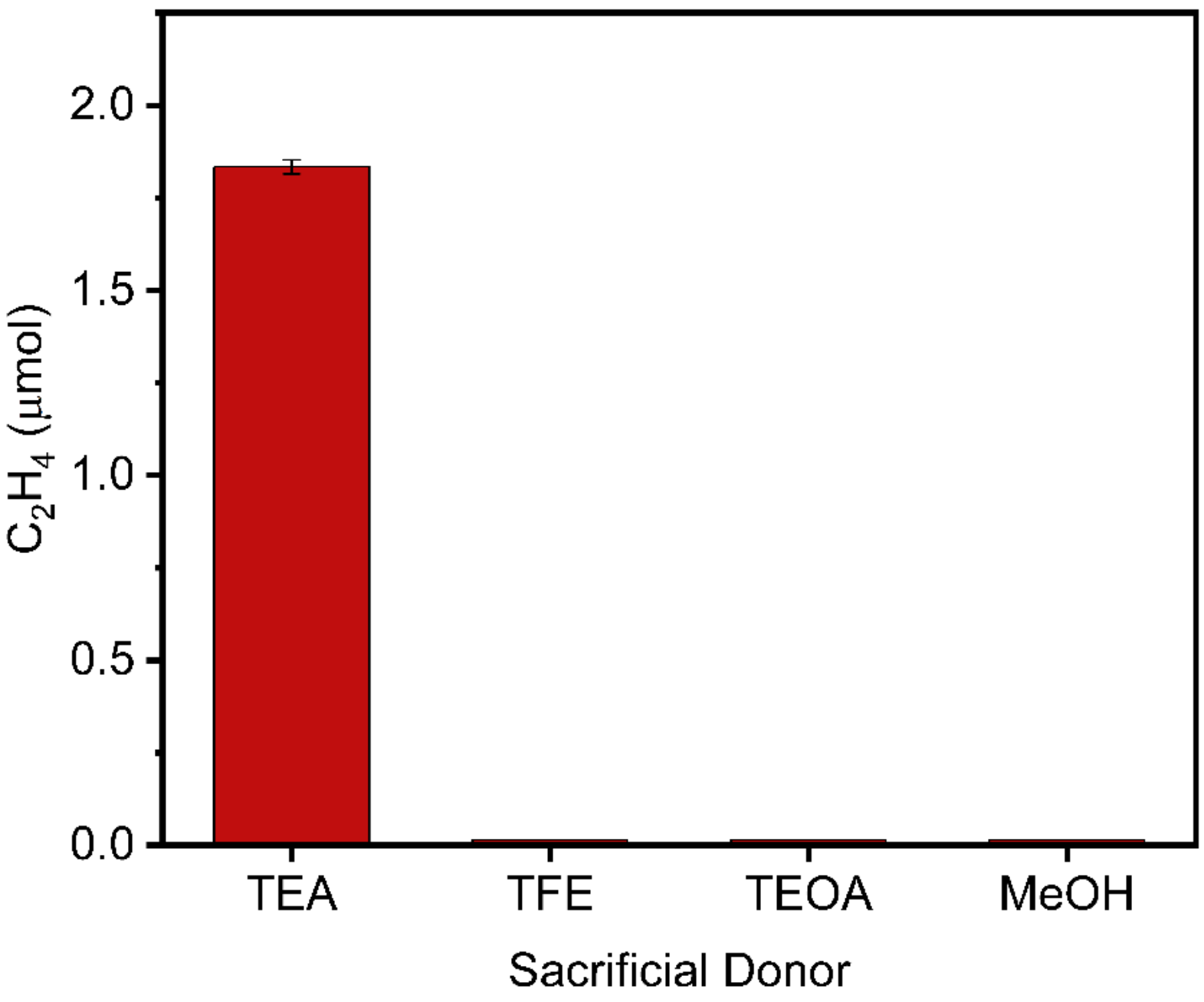

Figure S8. Amount of C_2H_4 (μmol) produced and selectivity (%) for C_2H_4 by the [Co(dmgH)$_2$(Im)Cl]/CuPS system containg 100 μM [Co(dmgH)$_2$(Im)Cl] and 250 μM CuPS in H_2O:ACN (1:1 *v*/*v*) mixture with 0.05 M of sacrifical donor under C_2H_2 (≥99.5 vol.%) after irradiation with 405 nm light (140 mW×cm^{-2}) for 4 h. Error bars indicate standard error of the mean, calculated from three runs.

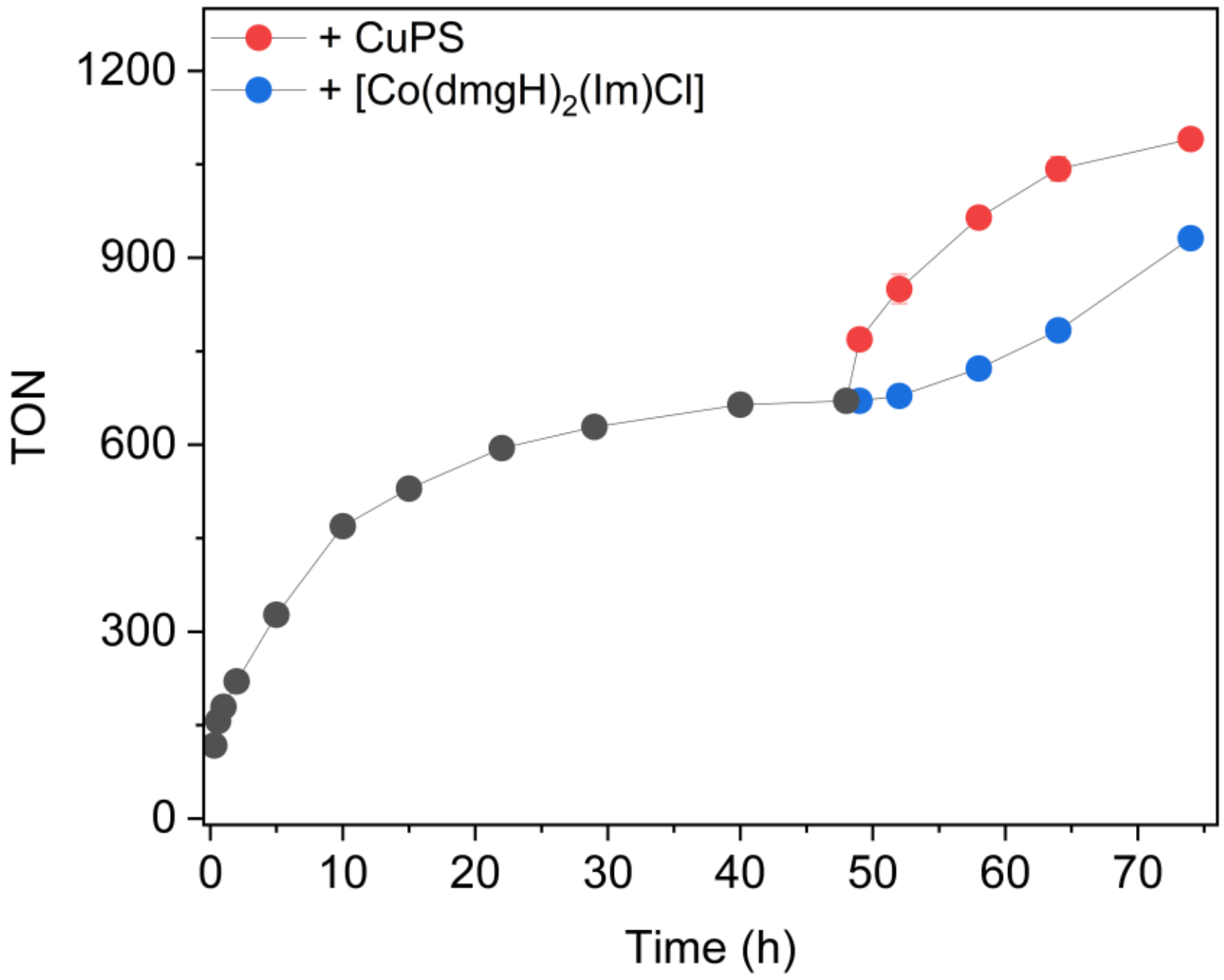

Figure S9. Recovery of TON (C_2H_4) upon addition of CuPS (250 µM, red circles) and [Co(dmgH)$_2$(Im)Cl] (100 µM, blue circles) to the solution containing [Co(dmgH)$_2$(Im)Cl] (100 µM), CuPS (250 µM) in H_2O:ACN (1:1 *v*/*v*) with 10% *v*/*v* TEA (pH = 11.0 ± 0.3) under 405 nm light (140 mW×cm^{-2}).

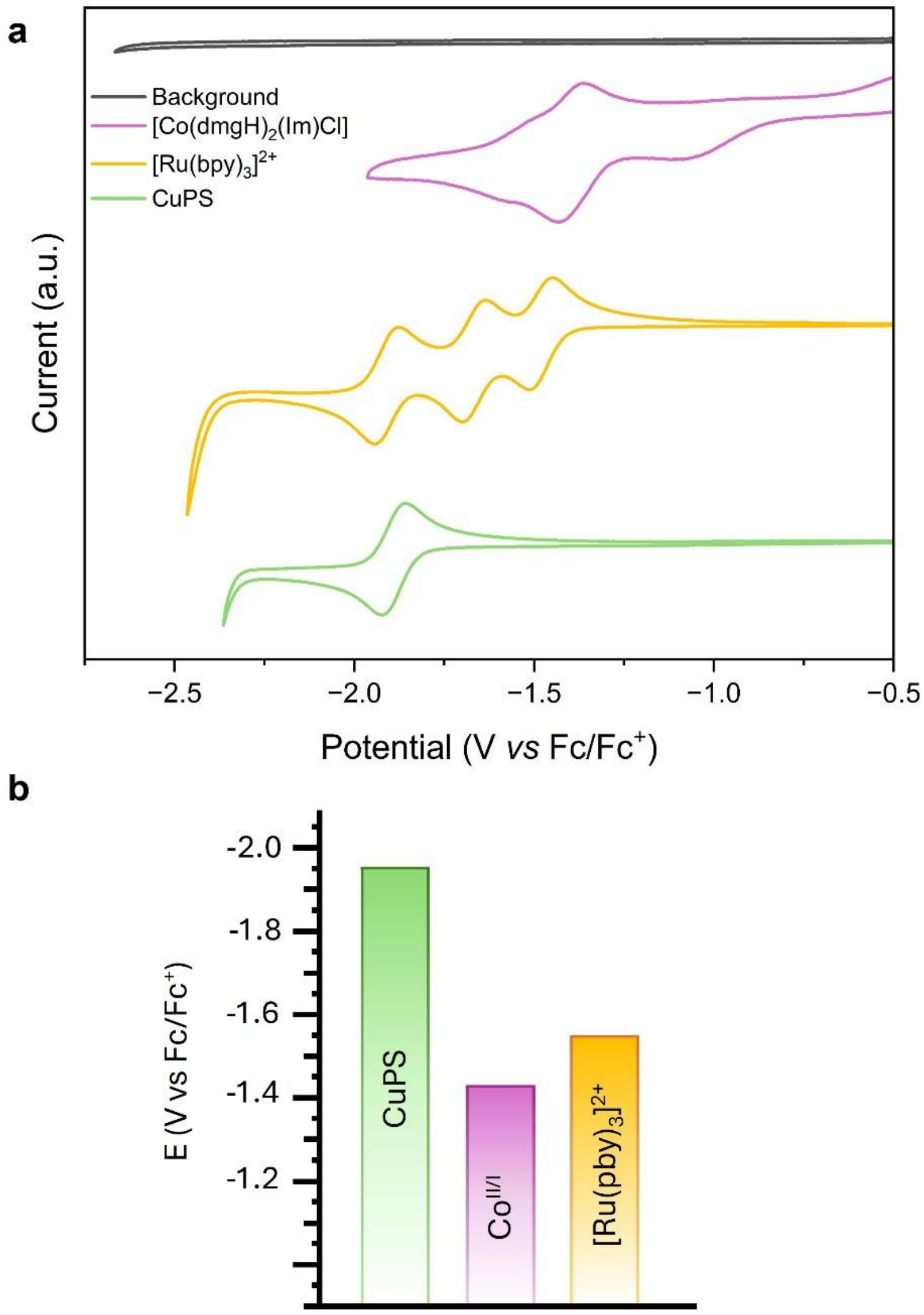

Figure S10. a) Electrochemical characterization of reduction potentials of CuPS (green, 1 mM), $[Ru(bpy)_3]^{2+}$ (orange, 1 mM), and $[Co(dmgH)_2(Im)Cl]$ (violet, 1 mM) in ACN at a scan rate of 100 $mV \times s^{-1}$ under N_2 using $TBAPF_6$ (0.1 M) as electrolyte. b) Comparison of reduction potential for CuPS, Co^{II}/Co^{I}, and $[Ru(bpy)_3]^{2+}$.

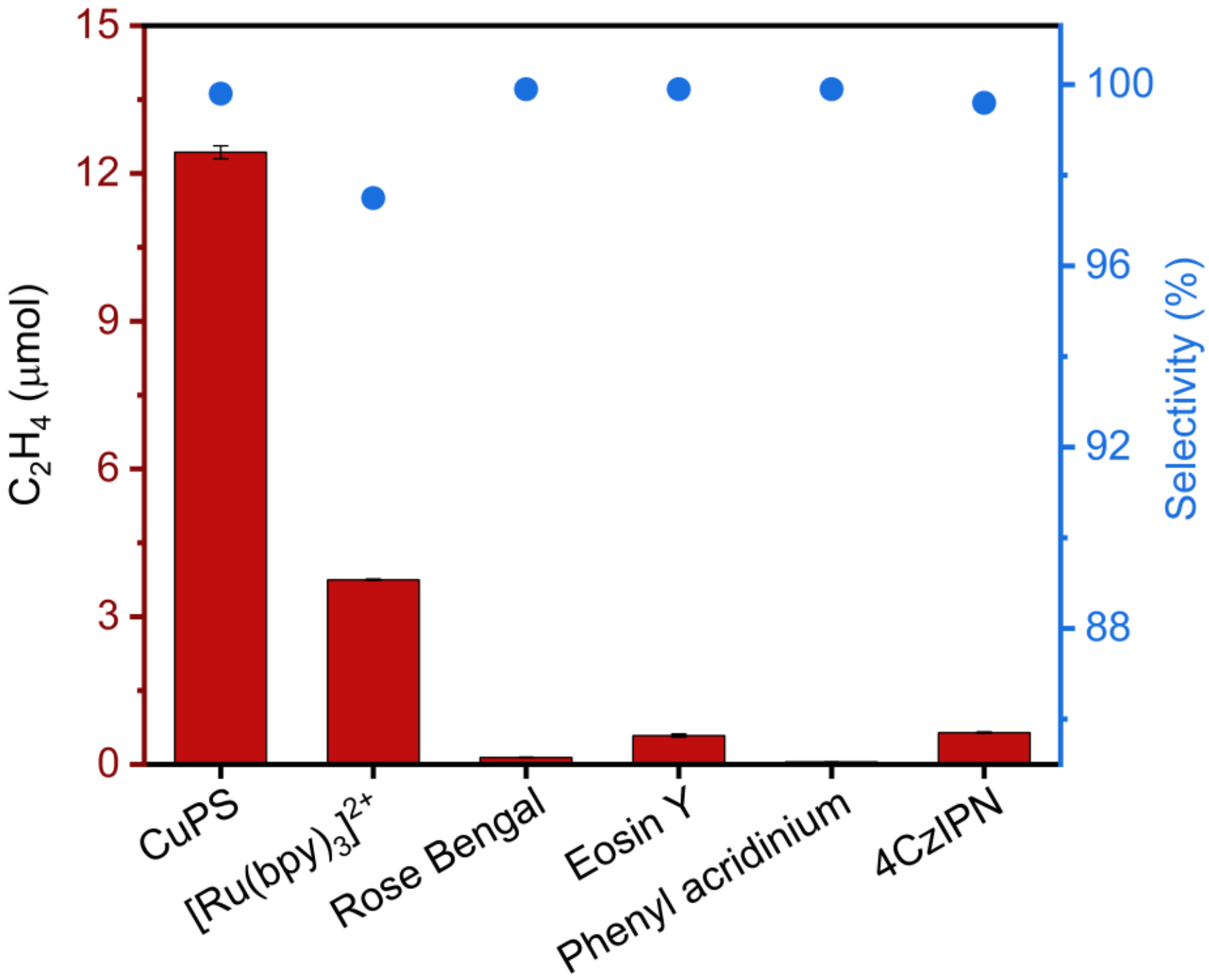

Figure S11. Amount of C_2H_4 (µmol) produced and selectivity (%) for C_2H_4 by the system containing 100 µM [Co(dmgH)$_2$(Im)Cl] and 250 µM of PS in H_2O:ACN (1:1 *v*/*v*) mixture with 10% *v*/*v* of TEA under C_2H_2 (≥99.5 vol.%) after irradiation at an intensity of 140 mW×cm^{-2} for 4 h. The CuPS-cointaing system was irradiated at 405 nm; the $[Ru(bpy)_3]^{2+}$- and 9-mesityl-3,6-di-*tert*-butyl-10-phenylacridinium tetrafluoroborate-containing systems were irradiated at 450 nm; the Rose Bengal-containg system was irradiated at 570 nm; the eosin Y-containing system was irradiated at 525 nm; the 4CzIPN-containing system was irradiated at 405 nm (we noted that a precipitate started forming after 90 min of irradiation). Error bars indicate standard error of the mean, calculated from three runs.

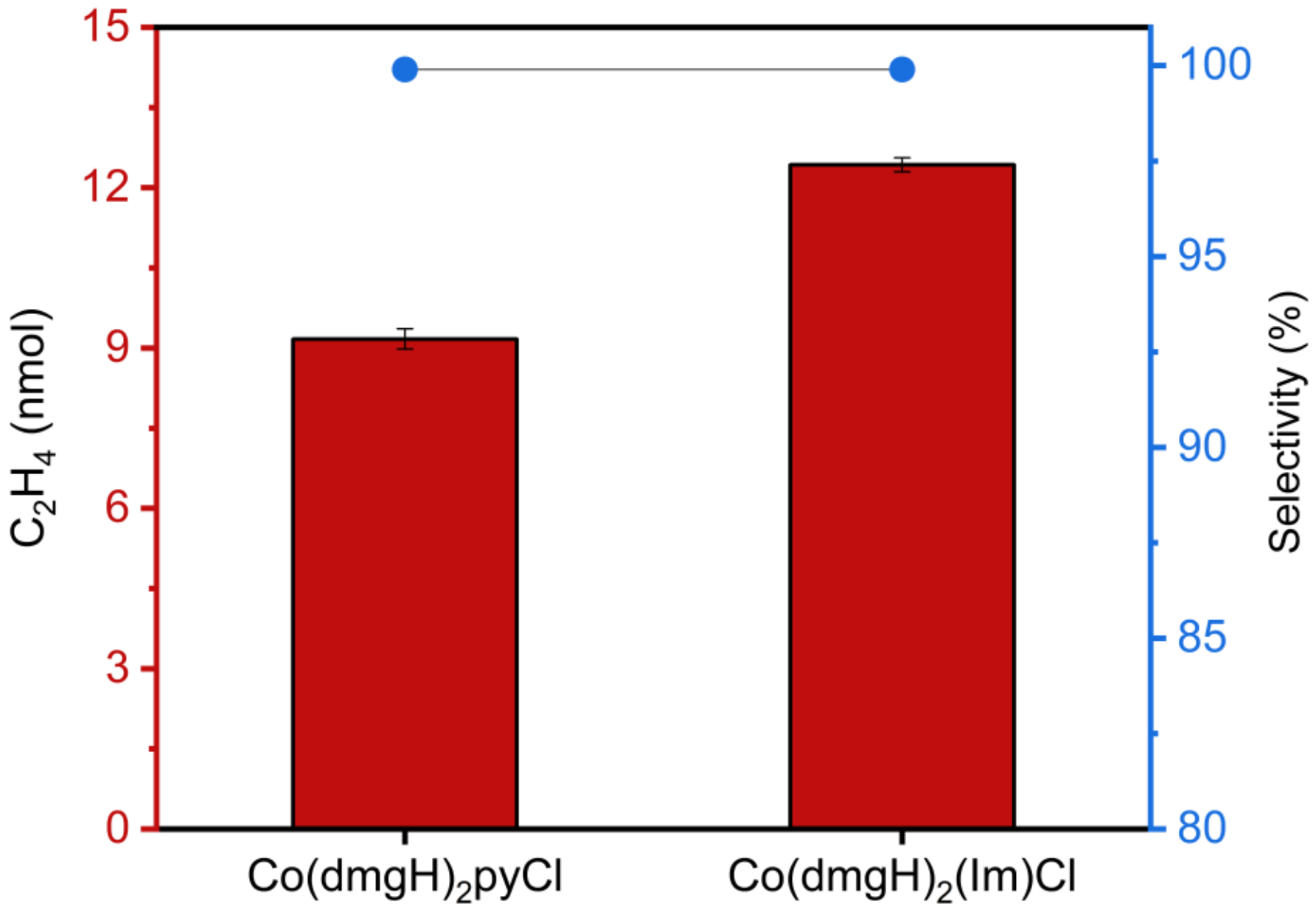

Figure S12. Amount of C_2H_4 (nmol) produced and selectivity (%) for C_2H_4 by the system containing 100 μM [Co(dmgH)$_2$(Im)Cl] and 250 μM CuPS in H_2O:ACN (1:1 *v*/*v*) mixture with 10% *v*/*v* TEA (pH = 11.0 ± 0.3) or the system containing 100 μM [Co(dmgH)$_2$pyCl] and 250 μM CuPS in H_2O:ACN (1:1 *v*/*v*) mixture with 10% *v*/*v* TEA (pH = 11.0 ± 0.3) under C_2H_2 (≥99.5 vol.%) after irradiation with 405 nm light (140 mW×cm^{-2}) for 4 h. Error bars indicate standard error of the mean, calculated from three runs.

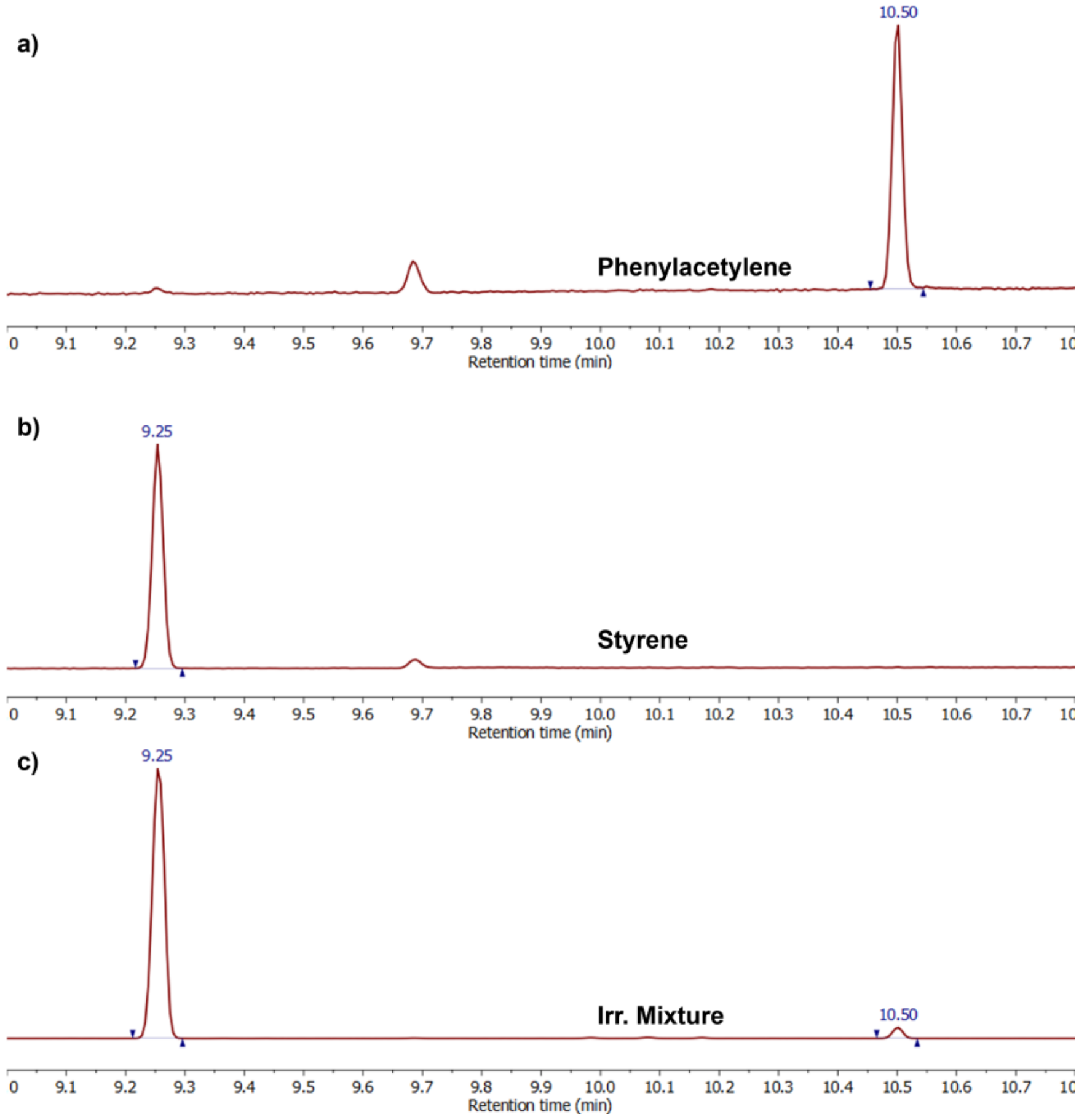

Figure S13. Gas chromatograms of a) phenylacetylene standard, b) styrene standard, c) and the photocatalytic mixture containing 10 mol% [Co(dmgH)$_2$(Im)Cl], 250 µM CuPS, and 10 mM phenylacetylene in H_2O:ACN (1:1 *v*/*v*) mixture with 10% *v*/*v* TEA (pH = 11.0 ± 0.3) after irradiation with 405 nm light (140 mW×cm^{-2}) for 4 h.

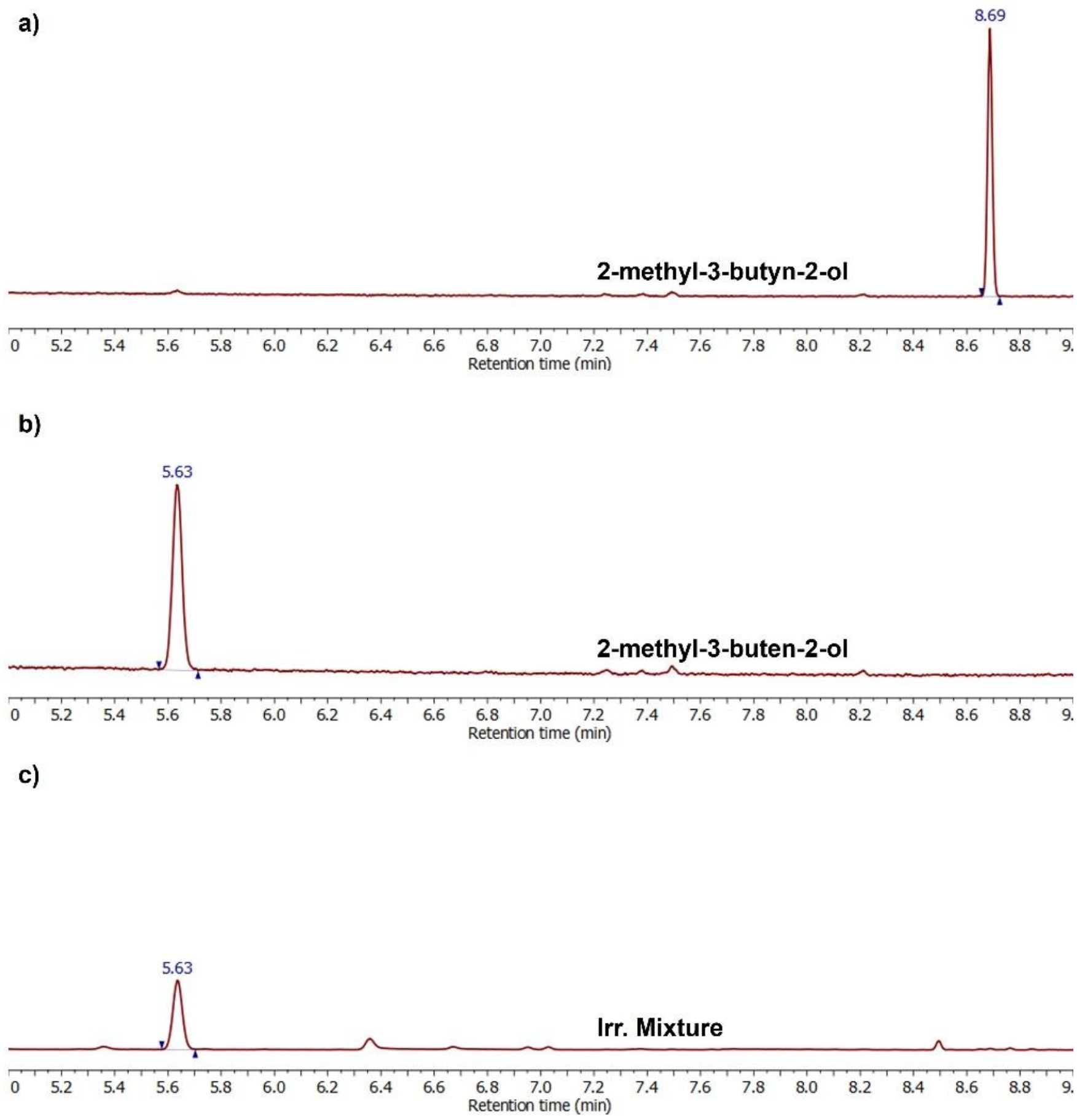

Figure S14. Gas chromatograms of a) 2-methyl-3-butyn-2-ol standard, b) 2-methyl-3-buten-2-ol standard, c) and the photocatalytic mixture containing 10 mol% [Co(dmgH)$_2$(Im)Cl], 250 µM CuPS, and 10 mM 2-methyl-3-butyn-2-ol in H_2O:ACN (1:1 *v*/*v*) mixture with 10% *v*/*v* TEA (pH = 11.0 ± 0.3) after irradiation with 405 nm light (140 mW×cm^{-2}) for 4 h.

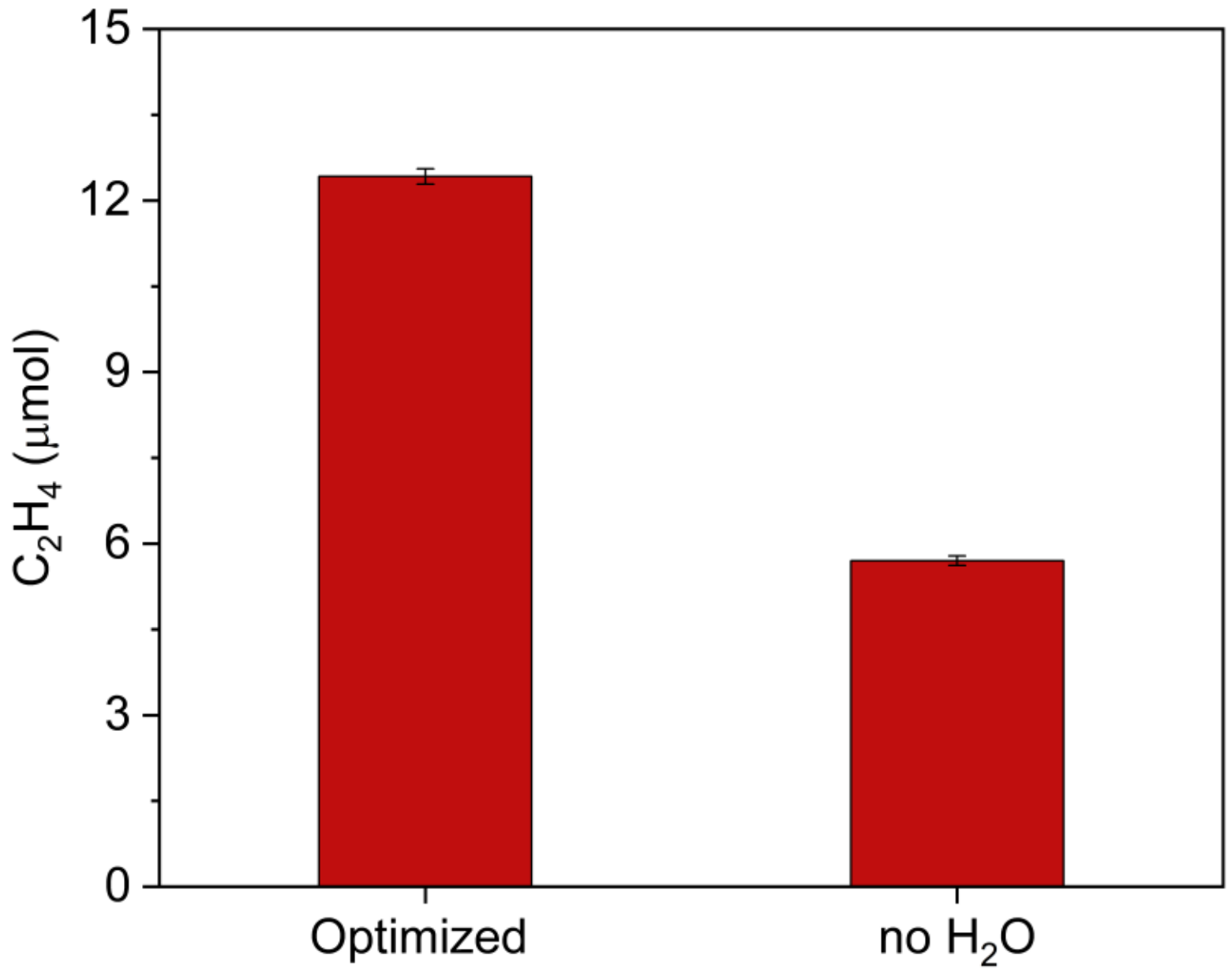

Figure S15. Amount of C_2H_4 produced for the optimized Co(dmgH)$_2$(Im)Cl]/CuPS system containing 100 μM [Co(dmgH)$_2$(Im)Cl] and 250 μM CuPS in H_2O:ACN (1:1 *v*/*v*) with 10% *v*/*v* TEA (pH = 11.0 ± 0.3) and the system containing 100 μM [Co(dmgH)$_2$(Im)Cl] and 250 μM CuPS in ACN with 10% *v*/*v* of TEA, irradiated 4 h at 405 nm (140 mW×cm^{-2}). Error bars indicate standard error of the mean, calculated from three runs.

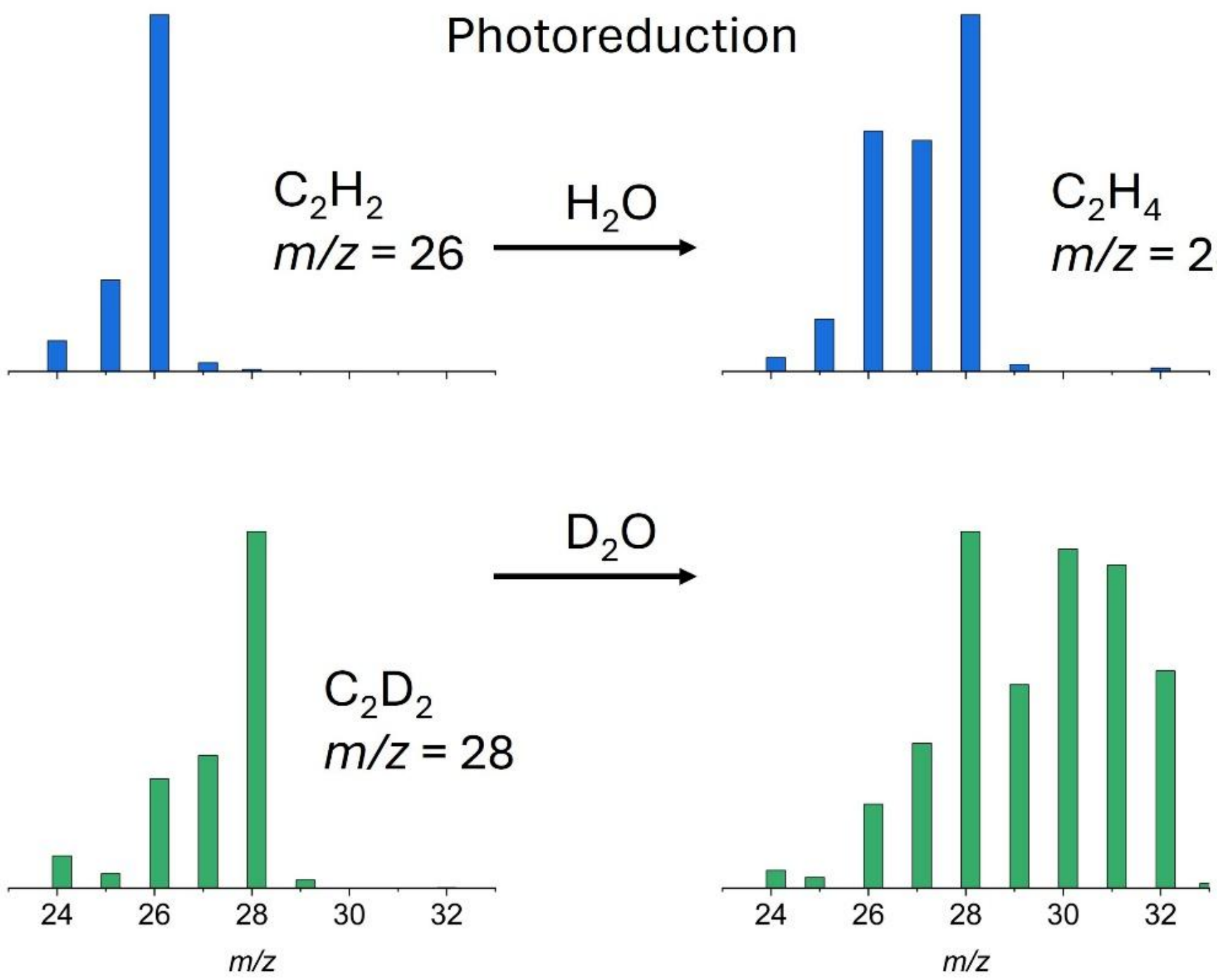

Figure S16. Mass spectra of acetylene (left) feedstock and ethylene (right) product for the irradiated (405 nm) [Co(dmgH)$_2$(Im)Cl]/CuPS system under C_2H_2 in MilliQ H_2O (blue) or in D_2O (green).

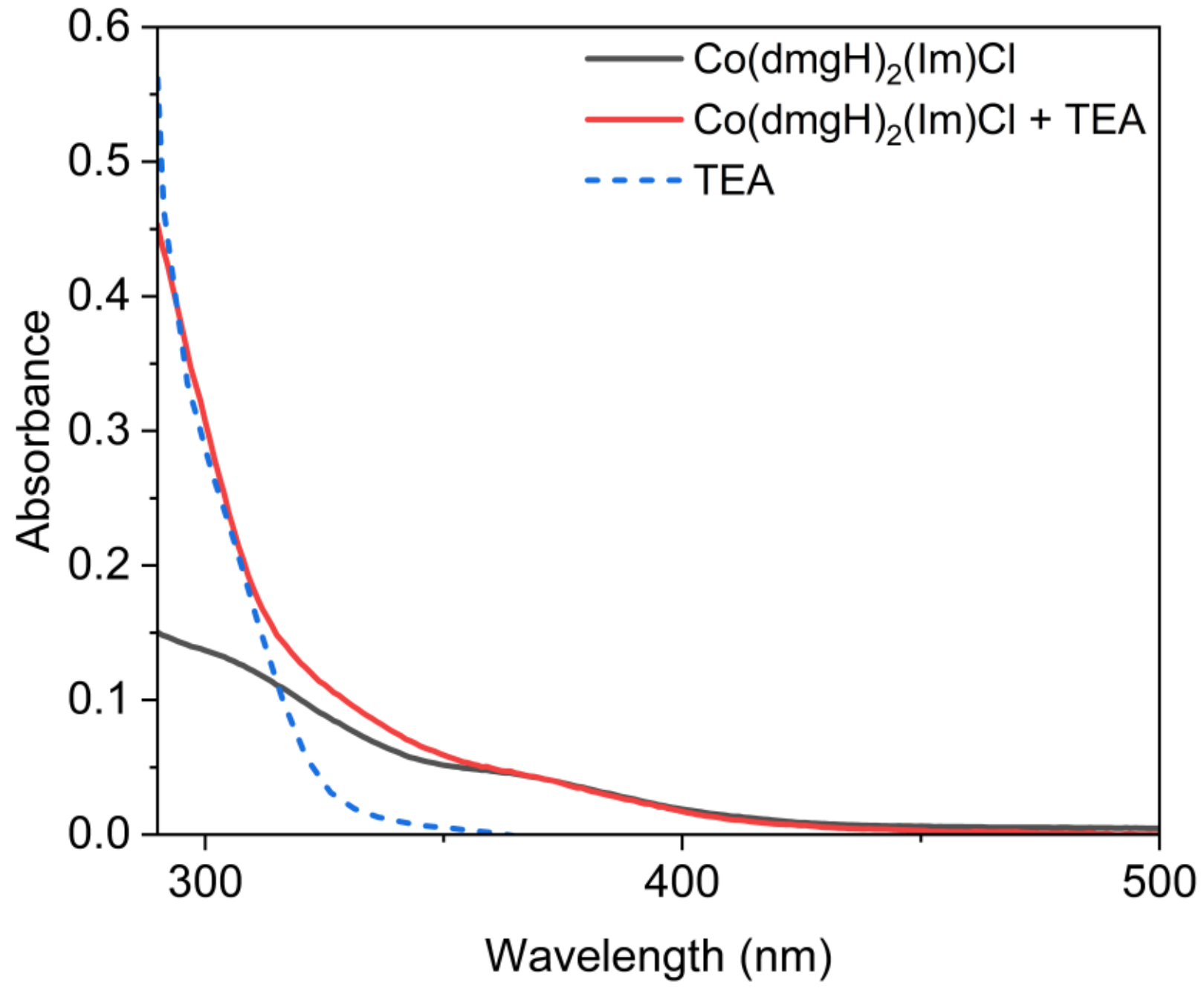

Figure S17. UV–vis spectra of the system containing 100 μM [Co(dmgH)$_2$(Im)Cl] in ACN with (red) or without (grey) TEA 10% *v*/*v*. Spectra of TEA 10% *v*/*v* in ACN is reporetd (dash line, blue).

Supplementary References

[1] F. Arcudi, L. Đorđević, N. Schweitzer, S. I. Stupp, E. A. Weiss, *Nat. Chem.,* 2022, **14**, 1007-1012.

[2] A. Panagiotopoulos, K. Ladomenou, D. Sun, V. Artero and A. G. Coutsolelos, Dalton Trans., 2016, **45**, 6732-6738.

[3] X. Zhang, M. Cibian, A. Call, K. Yamauchi, K. Sakai, *ACS Catal.,* 2019, **9**, 11263-11273.

[4] J. Osswald, K. Kovnir, M. Armbrüster, R. Giedigkeit, R. E. Jentoft, U. Wild, Y. Grin, R. Schlögl, *J. Catal,.* 2008, **258**, 219-227.
